# Tailoring the catalytic performance of MXenes in propane dehydrogenation by layer substitution from microkinetic simulations

**DOI:** 10.1016/j.isci.2025.113480

**Published:** 2025-09-07

**Authors:** Aqsa Abid, XiaoYing Sun, Yuqing Tang, Yi Xiao, Bo Li

**Affiliations:** 1 Shenyang National Laboratory for Materials Science, Institute of Metal Research, Chinese Academy of Sciences, ShenYang 110016, China; 2 School of Materials Science and Engineering, University of Science and Technology of China, ShenYang 110016, P.R. China; 3 Institute of Catalysis for Energy and Environment, College of Chemistry and Chemical Engineering, Shenyang Normal University, Shenyang 110034, China

**Keywords:** Chemical bonding, Chemical processing, Computational materials science, Simulation in materials science

## Abstract

The reactivity and catalytic performance of MXenes in propane dehydrogenation (PDH) are highly dependent on their composition. This study explores modifying MXenes by substituting their X-layers with carbon, nitrogen, or a mix of both. Density functional theory (DFT) and microkinetic simulations show that these substitutions substantially alter the catalytic mechanism. Nitrogen substitution shifts the *p*-band center of oxygen active sites closer to the Fermi level, significantly lowering the energy barrier for the first C−H bond activation. Microkinetic analysis further confirms that nitrogen-substituted MXenes exhibit the highest turnover frequency (TOF) for propane conversion and propylene formation. The findings demonstrate that PDH reactivity can be precisely tuned by X-layer substitution, offering a strategy for optimizing MXene-based catalysts.

## Highlights

• X-layer substitution (C, N, or C/N) tunes MXene geometric and electronic structure
• N-substituted MXenes effectively regulate p-band center of surface oxygen
• p-band center governs C−H activation barriers and enhances reactivity
• V_3_N_2_O_2_ MXene offer superior performance in catalytic propane dehydrogenation

## Graphical abstract

**Figure:**
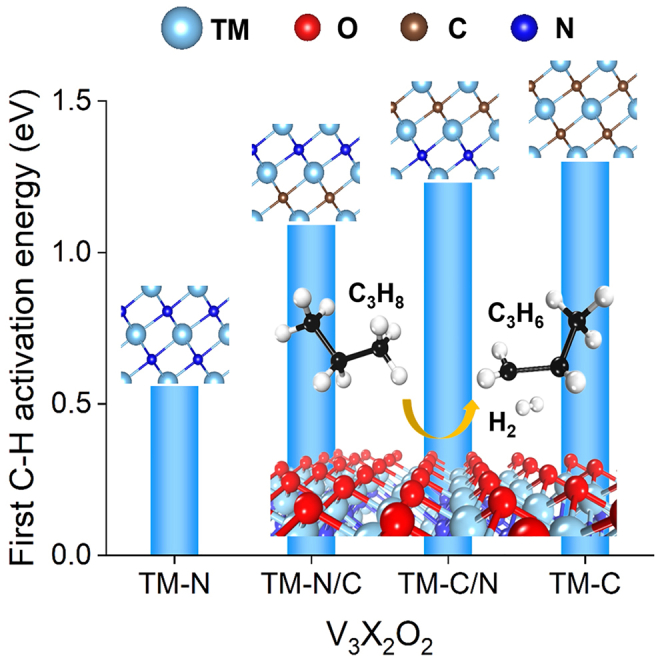

## Introduction

Propyleneis one of the major building blocks of a variety of petrochemical industrial products,^1^ including polypropylene, acrolein, acrylonitrile, propylene oxide, acetone, epoxy propane, etc.^2^^,^^3^^,^^4^ Conventional approaches for the production of propylene involve fluid catalytic cracking and steam cracking of naphtha as well as light diesel;^2^ however, these technologies are not selective toward propylene, which created a so-called propylene gap between demand and supply.^5^ The catalytic propane dehydrogeneration (PDH) is deemed to be the foremost approach for producing propylene with more than 80% selectivity toward the desired product.^2^^,^^6^ Moreover, evolution of hydraulic fracturing technology offers shale gas reservoirs rich with propane, thus, embark on achieving economical and abundant feedstock for the process of PDH.^7^ Catalytic dehydrogenation of propane is dominated by the choice of suitable catalyst. Conventional catalysts such as pure transition metals (TMs) like platinum and various TM oxides including vanadium oxide (VO_x_), molybdenum oxide (MoO_x_), and chromium oxide (CrO_x_) have been employed for this process owing to their high conversion and selectivity.^8^^,^^9^^,^^10^^,^^11^^,^^12^^,^^13^ On the other hand, metal- and metal-oxide-based catalysts are afflicted by the formation of carbonaceous species over catalyst surface which caused severe catalyst deactivation,^14^^,^^15^ and the required regeneration not only consumed operation time but also facilitated active species agglomeration. Therefore, it is highly urgent to identify catalysts that could overcome these challenges and offer improved catalytic performance.

MXenes are a class of two-dimensional (2D) TM-carbides, -nitrides, and -carbonitrides which are typically derived by selective etching of aluminum (Al) layer from their bulk correspondent (MAX phase) with the help of HF or LiF/HCl solution.^16^ The subsequent framework is impulsively functionalized with O, OH, or F and consequently generating X−M−O, X−M−OH, or X−M−F surface groups which in turn gives rise to MXenes (M_n+1_X_n_T_x_, where M denotes early transition metal, X represents C/N, and T refers to the terminal group). MXenes demonstrate distinguished properties due to a variety of functional groups which are considered to be the regulator of their electronic structure.^17^ Nonetheless, catalytic activity of MXenes is tremendously influenced by the type and distribution of the terminal group.^18^

Recently, Ti_3_AlC_2_ MAX phase has shown remarkable catalytic performance for oxidative dehydrogenation of n-butane to butenes and butadiene, wherein, a thin oxide surface layer exhibiting oxygen vacancies renders O-containing active sites.^19^ For O_2_/butane ratio of 0.25:1 at 550 °C, Ti_3_AlC_2_ shows up to 35% and 25% selectivities for butene and butadiene at 10% conversion. Moreover, when the proportion of oxygen is twice to that of the initial, the butane conversion becomes doubled without significant loss in product selectivity. Of importance, Ti_3_C_2_O_2_ MXene is found to be highly reactive for ethylbenzene dehydrogenation with reactivity about 92 μmol m^−2^ h^−1^ combined with lower energy barrier of 0.66 eV for the first C−H bond activation.^20^ Moreover, surface C−Ti−O groups serve as active sites, offering 97.5% selectivity toward styrene and 40 h stability with almost no catalyst deactivation. From computational exploration, Niu et al. presented a relationship between PDH catalytic activity and termination configuration of Ti_2_CT_2_ (T = O, OH) via quantitative descriptor known as the hydrogen affinity (E_H_).^21^ The E_H_ can be comprehended as an innate property of O and OH groups of Ti_2_C MXenes to abstract hydrogen from propane. Density functional theory (DFT) calculations reflected that it has a linear relationship with the statistical average of OH fraction and also correlated linearly with C−H activation energies. It is also revealed that MXenes with low hydrogen affinity (E_H_ < 1 eV) exhibit higher activity toward C−H bond activation on methylene group (−CH_2_−) of propane. In the follow-up work, Niu and coworkers observed that the mean hydrogen affinity of Ti_2_CT_2_ (T = O, OH, and F) MXene corresponds linearly with overall ratio of O functional group (x_O_) such that the hydrogen affinity increases as x_O_ decreases, irrespective to the species of surface terminations.^22^ Furthermore, the influence of F termination on the hydrogen affinity is weaker than that of OH termination over same O ratio. This is due to the fact that the O- and F-terminated Ti_2_C possesses lower hydrogen affinity than that of O and OH terminated.

While the surface chemistry of MXenes is pivotal in dictating their properties and catalytic efficiency, their layered structure offers more flexibility; particularly the “X” layer, composed of carbon or nitrogen, can be effectively modified to tailor the intrinsic properties of MXenes, such as their fundamental structure, electronic attributes, and chemical reactivity, which is essential for optimizing the catalytic process in PDH. It might offer more advantages than merely modifying the surface oxygen layer in a controllable manner, potentially leading to superior catalyst activity and selectivity.

Herein, we have thoroughly investigated the effects of varying the X-layer composition in a series MXenes (M_3_X_2_O_2_, M = Ti, V, Nb, Mo, or Ta) for their catalytic performances in PDH reaction by a combination of DFT and microkinetic simulation. It is noted that some investigated MXenes in current work have been successfully synthesized experimentally which corroborated the viability of chosen configurations.^23^^,^^24^^,^^25^^,^^26^^,^^27^^,^^28^^,^^29^

## Results and discussion

### MXene structural models

**Figure 1:**
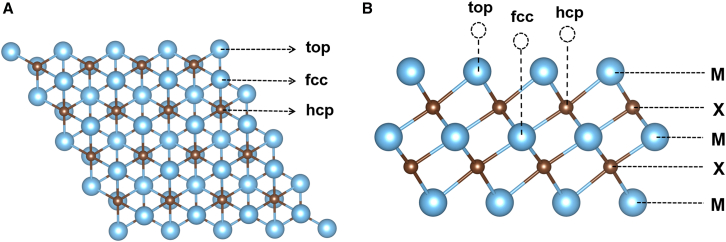
Structural models of double-layer MXenes (A) Top view. (B) Side view.

A 4 × 4 supercell of double layer MXenes (M_3_X_2_T_2_, where M = Ti, V, Nb, Mo, or Ta; X = C, N, or CN) is used as catalyst model as shown in Figure 1A. Moreover, the possible sites including top, face-centered cubic (fcc), and hexagonal close-packed (hcp) are explored in the calculation. It is noted that there are two X layers as shown in Figure 1B, and therefore, the substitution can be pure carbon or nitrogen, or carbon and nitrogen mixed which resulted in total four X-layer substitution models as shown in Figure S1. The layer compositions are designated as TM-C (pure carbon), TM-N (pure nitrogen), TM-C/N (mixed carbon-over-nitrogen), and TM-N/C (mixed nitrogen-over-carbon), where the notation explicitly indicates the vertical stacking sequence along the c-axis. Specifically, in TM-C/N, the upper layer is carbon with nitrogen beneath, while in TM-N/C, the upper layer is nitrogen with carbon beneath, as indicated in Figure S1. Atomic coordinates for representative structures are provided in Table S1.

Chemically reactive bare MXenes are prone to be functionalized with surface groups; among these, oxygen-terminated MXenes are thermodynamically favorable.^30^^,^^31^^,^^32^ Moreover the terminal oxygen species of MXenes are widely recognized as the active site in PDH.^31^ Therefore, MXenes including TM-C, TM-N, TM-C/N, and TM-N/C are functionalized with terminal oxygen by considering anchoring sites of the various combinations of fcc, hcp, and top sites, which is then used as the catalyst model in PDH as illustrated in Figure S2.

### Formation energy and thermodynamic stability

The relative stability of these catalysts is evaluated as shown in Figure S3, and the most stable configurations for each investigated MXenes are indicated in Table S2. The calculations revealed that fcc-fcc configuration is most stable for the majority of TM-C and TM-N, whereas fcc-hcp is preferred for most of TM-C/N and TM-N/C. Furthermore, the formation energy (ΔE_f_) is consistently negative, indicating that all these MXenes are thermodynamically stable relative to their constituent elements, as shown in Figure S4.

### Geometric effects of X-layer substitution

**Figure 2:**
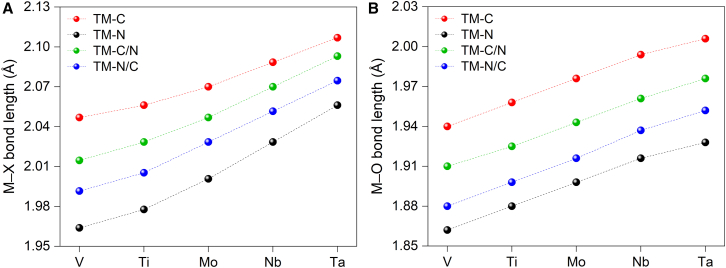
Bond length variations in MXenes after X-layer substitution (A) M−X bond lengths. (B) M−O bond lengths.

Structural analysis of the optimized MXene catalysts reveals that the X-layer substitution simultaneously affects both M−X and M−O bond distances, as shown in Figure 2. The M−X bonds are consistently ∼0.1 Å longer than corresponding M−O bonds for all investigated catalysts, and the bond distance of both M−X and M−O is in an increasing order of V, Ti, Mo, Nb, and Ta, which implied that vanadium might has the strongest bonding among investigated metals. For each MXene catalyst, the M−X and M−O bond distance increases in the order of TM-N, TM-N/C, TM-C/N, to TM-C. For the M−X bond, the difference among the four X-layer substitution catalysts gradually decreases along the series of V, Ti, Mo, Nb, and Ta, while for the M−O bond, the difference remains nearly unchanged.

### Regulation of catalytic reactivity

**Figure 3:**
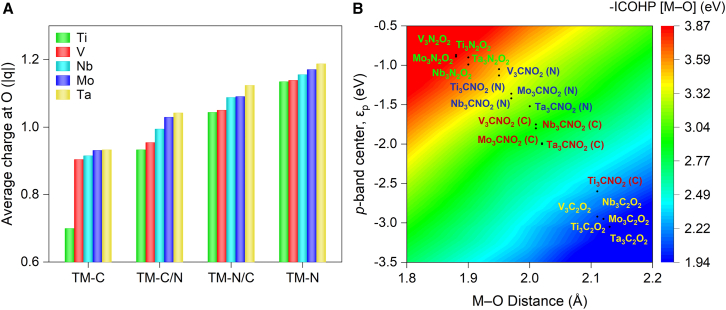
Electronic and geometric effects of X-layer substitution (A) Charge distribution. (B) Correlation between M−O bond properties and *p*-band center.

Modifying the X component in MXenes affect not only the geometry but also electronic structure as revealed from projected density of states analysis shown in Figure S4. As evident from Figure S5, all MXenes exhibit a high density of states near Fermi level which is contributed by terminal O atoms and sublayer metal atoms. The contributions are predominantly from the p-orbitals of O atoms at Fermi level, which explains the better reactivity of oxygen. However, in Mo_3_C_2_O_2_ (TM-C) and Ti_3_CNO_2_ (TM-N/C), the d-orbitals of metals contribute slightly greater at Fermi level than the p-orbitals of terminal oxygen atoms. The X-layer effects are also clearly manifested in the charges associated with surface oxygen obtained from Bader analysis as shown in Figure 3A, which showed a decreasing order along TM-N, TM-N/C, TM-C/N, and TM-C, and oxygen on vanadium-based MXenes possessed the most charges while oxygen on tantalum has the least charges among all investigated catalysts. It is clearly indicated that the nitrogen layer (TM-N and TM-N/C) caused more charges transferred to surface oxygen than carbon layer (TM-C/N and TM-C).

Furthermore, the investigated MXene catalysts are categorized as four groups according to X-layer component which are closely related with both M−O bond distance and strength, and effectively regulated *p*-center of surface oxygen as shown in Figure 3B. It is found that the layer substitution moves the *p*-band center of surface oxygen closer to the Fermi level in the order of TM-N, TM-N/C, TM-C/N, and TM-C. This movement enhances the MXene reactivity by enabling facile electron transfer between terminal oxygen and reactant. Meanwhile, the M−O bond strength increases and the M−O bond length decreases. Therefore, it is clearly indicated the X-layer substitution effectively changed both geometry and electronic structure of MXene catalyst, which consequently adjust the reactivity. From above analysis, it is suggested that nitrogen layer could bring better reactivity than carbon layer.

### PDH mechanism on MXenes

**Scheme 1:**
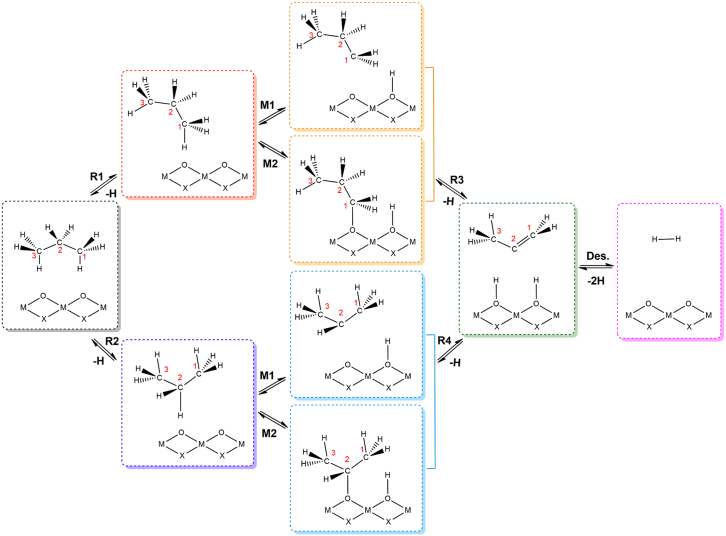
Schematic representation of reaction pathway for PDH reaction

The dehydrogenation of propane to form propylene has been well recognized to occur via successive removal of two hydrogen atoms from a propane molecule. Hydrogen atoms can be further categorized as primary hydrogen bonded with terminal methyl group (−CH_3_) and secondary hydrogen linked with middle methylene bridge (−CH_2_−) of a propane molecule. For this reason, two routes are available that could be initiated from either primary hydrogen (1-C_3_H_7_) or secondary hydrogen (2-C_3_H_7_). The whole reaction network incorporates propane adsorption, first and second hydrogen elimination, propylene formation, and propylene and hydrogen desorption as shown in Scheme 1. Initially, propane (C_3_H_8_) molecule is physically adsorbed on the surface of MXenes at a distance of 3 Å, with an adsorption energy of around −0.2 eV (Figure S6). Either primary or secondary hydrogen is pointing to the surface oxygen site as the preliminary step referred as R1 and R2 for the following hydrogen abstraction. At next step, the scission of the first C−H bond in propane leads to the formation of a propyl radical (M1) or an adsorbed propyl (M2), respectively, and a surface hydrogen (H∗). The second C−H bond activation results in a successive removal of another hydrogen atom from propyl specie with the formation of propylene (C_3_H_6_), termed as R3 and R4. The product can be readily desorbed from the surface of catalyst. Recombination of both hydrogen atoms from active sites produces hydrogen molecule (H_2_), thus regenerating active sites.

**Figure 4:**
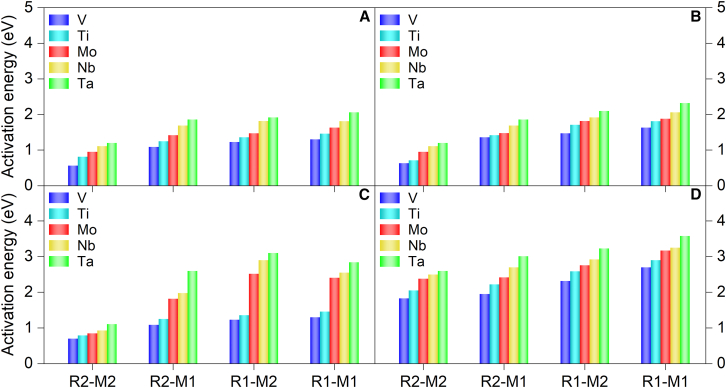
Activation energies for the first C−H bond cleavage in propane (A−D) (A) TM-N, (B) TM-N/C, (C) TM-C/N, and (D) TM-C MXenes, following pathways R2-M2, R2-M1, R1-M2, and R1-M1, respectively, as outlined in Scheme 1.

The nature of X-layer, type of TM, and the route of reaction significantly influence the catalytic activity of MXenes for PDH process, as given in Figure 4. For the first dehydrogenation step, the lowest energy barriers are achieved by TM-N catalysts, indicating that nitrogen as X-layer is catalytically more active, and this observation is consistent with previous electronic structure analysis. Notably, vanadium-based MXenes showed relatively lower activation energies compared with the other metals, indicating their potential as a good candidate for PDH catalysts. Moreover, the reaction proceeding along R2-M2 imparted relatively lower energy barriers than other reaction pathways because of potentially more stabilized transition state for cleaving the C−H bond, as illustrated by and Figures S7 and S8. The lowest energy barrier for the first C−H bond activation along R2-M2 is calculated to be 0.56 eV from V_3_N_2_O_2_. Besides, Mo_3_N_2_O_2_ and Ti_3_N_2_O_2_ also manifest optimal catalytic activity for the same reaction pathway, exhibiting the energy barrier of less than 1 eV. The energy barriers of all the nitrogen-substituted MXenes, M_3_N_2_O_2_ (M = Ti, V, Nb, Mo, or Ta), are significantly lower than those of conventional metal oxide catalysts, such as Cr_2_O_3_ (1.37 eV) and VO_x_/Al_2_O_3_ (∼1.2 eV).^10^^,^^33^

On the other hand, the second C−H bond activation energy is smaller than the counterpart of first C−H bond activation for all investigated catalysts, as given by Figure S9, which confirmed that first C−H bond activation is the most difficult step on pathway. Moreover, the trend of second C−H bond activation is similar to the first C−H bond activation, that TM-N exhibited lower barriers compared to TM-C and TM-C/N or TM-N/C. The activation energies for both first and second C−H bond cleavage include zero-point energy corrections, calculated from vibrational frequencies for each reaction pathway, as given in Table S3.

**Figure 5:**
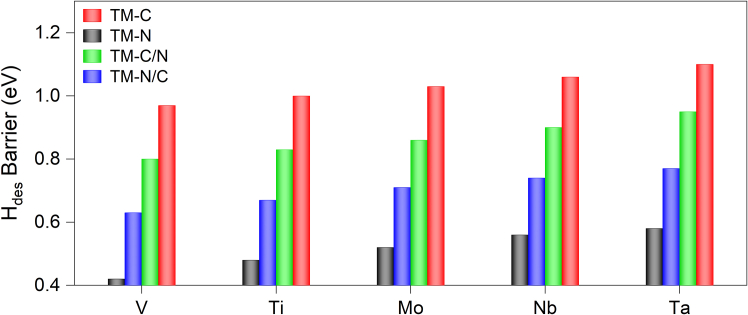
Comparison of hydrogen desorption barrier on TM-C, TM-N, TM-C/N, and TM-N/C with TM = V, Ti, Mo, Nb, and Ta, respectively

The hydrogen atoms bound to the surface, which are abstracted from propane during the first and second dehydrogenation steps, must desorb from the surface to complete the catalytic cycle. For this reason, both surface-bound hydrogen atoms recombine and are released as a hydrogen molecule. The calculated energy barriers for H_2_ formation on TM-C, TM-N, TM-C/N, and TM-N/C are shown in Figure 5. For all the catalysts, the energy barrier for hydrogen molecule formation lies between the barriers of the first and second C−H bond activation. The hydrogen desorption barrier for V_3_N_2_O_2_ (0.42 eV) is the lowest which is markedly lower than the ∼1.2 eV reported for Pt catalysts,^34^ demonstrating the advantage of nitrogen-substituted MXenes in reducing energetic bottleneck for hydrogen removal. Notably, across all investigated catalysts, the trend of the hydrogen molecule formation barrier is similar to that of the first and second C−H bond activation, that the barriers are the lowest for TM-N and highest for TM-C. This similarity indicates that the *p*-band center of oxygen plays a dominant role not only in C−H bond activation but also in hydrogen molecule formation, likely due to its influence on the electronic structure and reactivity of the catalyst surface. The nature of the transition metal, in addition to the substituted layer, significantly influences the hydrogen desorption barrier. Among the transition metals, V exhibits the lowest hydrogen desorption energy barrier. The barriers then increase in the order of Ti, Mo, Nb, and Ta.

**Figure 6:**
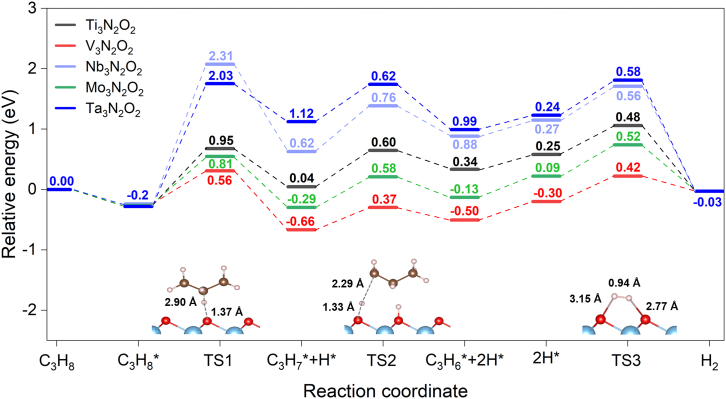
Potential energy profile and the associated transition states of PDH reaction via R2-M2-R4

Figure 6 illustrates the most favorable reaction pathway (R2-M2-R4) for TM-N catalysts, and considering both first and second C−H bond activation barriers, V_3_N_2_O_2_ emerges as the optimal catalyst. Additional calculations examining deep dehydrogenation pathways on V_3_N_2_O_2_ reveal significantly high energy barriers (>1.8 eV) for reactions beyond propylene formation, as shown in Figure S10. These results suggest that the catalyst effectively suppresses undesired side reactions. Regarding the mechanism of C−H bond activation, it is found that the surface oxygen atoms donate electron to the anti-bonding orbital of propane molecule, thus weakening the C−H bond as shown in Figure S11.

**Figure 7:**
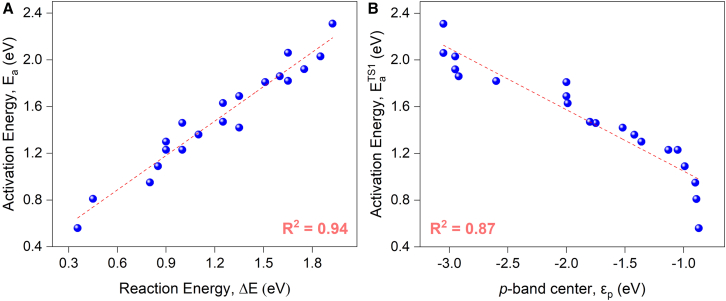
Scaling relationships in propane dehydrogenation (A) BEP relationship. (B) *p*-band center vs. activation energy.

More interestingly, it is found that Brønsted-Evans-Polanyi (BEP) relation is valid for the first C−H bond activation, which is deemed as the most difficult step on the pathway; in detail the reaction energy showed a good linear relation with the calculated barrier as shown in Figure 7A, and lower reaction energy leads to smaller barrier. On the other hand, the *p-*band center of surface oxygen also held a linear relation with the first and second activation barriers, as well as the hydrogen desorption barrier, which is somehow expected, as given in Figures 7B and S12. These consistent relationships emerge because the *p*-band center governs the nucleophilicity of surface oxygen sites, as it shifts closer to the Fermi level which is conductive for dehydrogenation.^35^ More profoundly, it is noted that X-layer substitution strategy effectively adjust the *p*-band center as shown in Figure 3B, therefore a direct link between X-layer substitution and C−H bond activation is established via *p*-band center of surface oxygen.

### Microkinetic studies on PDH performance

**Figure 8:**
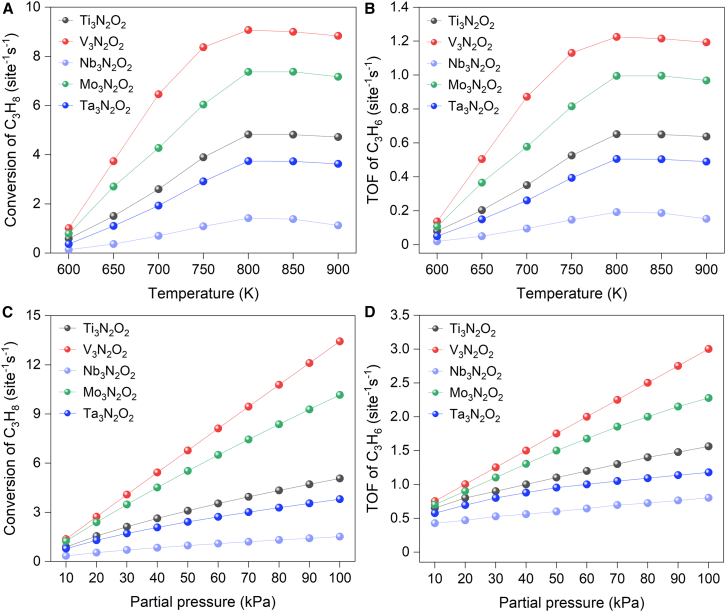
Temperature- and pressure-dependent TOFs for propane dehydrogenation (A and B) Propane conversion TOF (A) and propylene formation TOF (B) as a function of temperature. (C and D) Propane conversion TOF (C) and propylene formation TOF (D) as a function of partial pressure at 800 K.

To corroborate the predictions from potential energy surface calculations, microkinetic modeling simulations are performed to reveal the kinetic features of PDH reaction. Based on available pathways (given in Scheme 1), the elementary steps involved in the dehydrogenation of propane are listed in Table S4. Turnover frequency (TOF) of both propane conversion and propene formation showed a strong dependence on temperature as shown in Figures 8A, 8B, and S13. It is noted that the TOF reached the highest value at around 800 K which is optimum temperature for reaction. Among all investigated catalysts, the catalysts containing nitrogen layers have the largest TOF for both propane conversion and propene formation, which further verified the promotional effects induced by the nitrogen layer. Further analysis reveals that the increasing temperatures promote molecular activation, leading to a characteristic decrease in apparent activation energy as shown in Figure S14. Notably, TM-N catalysts demonstrate substantially lower apparent activation energies than TM-C, TM-C/N, and TM-N/C. Across all catalyst systems, this decrease becomes increasingly significant at higher temperatures, which are reduced to 25% of their initial values at 800 K.

At 800 K, increasing the propane partial pressure further enhances the catalytic performance of MXenes as shown in Figures 8C, 8D, and S15. This enhancement occurs because the higher pressure leads to a higher concentration of propane molecules on the catalyst, which in turn results in more frequent collisions with active sites. This improvement in C_3_H_8_ conversion and C_3_H_6_ formation TOF is more prominent for TM-N catalysts.

**Figure 9:**
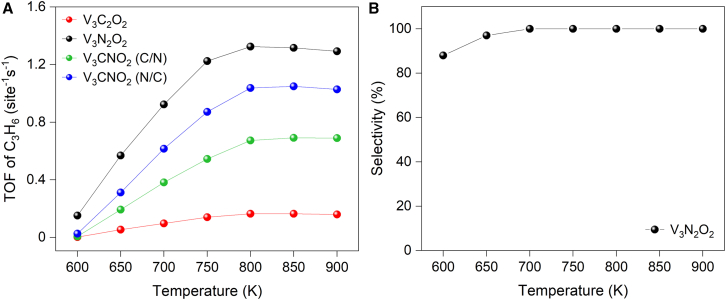
Catalytic performance of V-based MXenes (A) TOF comparison. (B) Temperature-dependent propylene selectivity of the best performed V_3_N_2_O_2_.

For the best performed vanadium-based catalysts, the X-layer effects are further explored as shown in Figure 9A. First of all, the largest TOF of propylene formation is obtained for V_3_N_2_O_2_ which is even better than the platinum.^34^ Furthermore, the performance sequence of TOF is at a decreasing order of TM-N, TM-N/C, TM-C/N, and TM-C which is consistent with the predictions from the calculated barrier on reaction pathway. More importantly, this trend is applied for all investigated MXene catalysts as shown in Figure S16. Therefore, TOF calculations further verified that the X-layer substitution is an effective method to adjust the catalytic performance.

The contribution of various pathways to propylene formation has been analyzed via reactant flux calculations from microkinetic simulations. As shown in Figure S17, the R2-M2 (2-C_3_H_7_∗) pathway dominates, accounting for 50% of the total propylene formation rate for the best-performing catalyst, V_3_N_2_O_2_, while the remaining pathways (R1-M1, R1-M2, and R2-M1) contribute less significantly. This preference aligns with the lower activation energy barrier via secondary hydrogen abstraction (R2-M2), as demonstrated in the potential energy profiles.

To further elucidate the role of individual elementary steps in the overall reaction kinetics, the degree of rate control (DRC) was calculated. The DRC coefficients (X_RC_) for each elementary step across TM-C, TM-N, TM-C/N, and TM-N/C are shown in Figures S18 and S19. Notably, the first C−H bond activation via R1-M1 pathway exhibits the highest X_RC_ for all catalysts, with values approaching unity in most cases, confirming its dominance as the rate-determining step. This aligns with the earlier findings from activation energy barriers. Furthermore, nitrogen-substituted MXenes (TM-N) show a lower X_RC_ for the first C−H activation step compared to TM-C, TM-C/N, and TM-N/C, indicating a reduced kinetic sensitivity to this step. This trend correlates with their lower activation barriers and higher TOFs, suggesting that nitrogen substitution not only enhances activity but also mitigates the kinetic bottleneck imposed by the rate-determining step.

V_3_N_2_O_2_ not only exhibits the highest TOF for propylene formation but also demonstrates strong resistance to deep dehydrogenation, effectively suppressing undesired side reactions. The combination of low activation barriers for PDH and high barriers for deep dehydrogenation explains the observed high selectivity. Figure 9B reveals that the propylene selectivity is temperature-dependent, where V_3_N_2_O_2_ exhibits reduced selectivity at lower temperatures (600–650 K). However, as the temperature increases beyond 650 K, side reactions are significantly suppressed, leading to 100% selectivity. The nitrogen-substituted V_3_N_2_O_2_ MXene demonstrates markedly higher catalytic activity in PDH than experimentally reported catalysts, including metal oxides, MAX phases, and carbide-based MXenes, as quantified in Table S5.^10^^,^^19^^,^^20^^,^^33^

### Conclusion

In the current work, a combined DFT and microkinetic simulation is performed to calibrate the catalytic effects of X-layer substitution for a series of MXene catalysts in PDH. Nitrogen, carbon, or mixed layer substitution are explored at the same footing which provide unbiased evaluation of X-layer effects. It is found that X-layer substitution significantly affected both geometry and electronic structure of MXene catalysts. Both distance and strength of M−O bond are well categorized according to the component of X-layer, and *p*-center of surface oxygen is also effectively adjusted by varying X-layer. The PDH proceeding from secondary-to-primary (R2) hydrogen abstraction following the co-adsorption of hydrogen and propyl radical on the active sites (M2) renders lower C−H bond activation energy compared with other pathways. More importantly, MXene with nitrogen layer possessed the lowest C−H bond activation barrier, and the calculated barrier is in an increasing order of TM-N/C, TM-C/N, and TM-C for the rest. Therefore, X-layer substitution is proven to be effective to tune C−H bond activation ability. Moreover, the calculated C−H bond activation barrier obeyed BEP relation and showed a good linear relation with the *p*-center of surface oxygen. The calculated TOF from microkinetic simulation further corroborated the effects of X-layer substitution which demonstrate an activity trend in a decreasing order of TM-N, TM-N/C, TM-C/N, and TM-C, and is consistent with the C−H bond activation results. In the end, both reaction pathway and microkinetic simulation identify that V_3_N_2_O_2_ has the best performance among the investigated MXene catalysts and even exceeded the conventional Pt catalysts. Overall, the current work not only highlights the great potential of MXene as catalyst for PDH but also confirms the tunable effects aroused by X-layer substitution, which paves the way for further rational improvements.

### Limitations of the study

While this work provides insights into the catalytic performance of X-layer-substituted MXenes for PDH, several limitations should be noted. First, the study relies on idealized models of MXenes (e.g., perfect oxygen termination and uniform layer substitution), which may not fully capture the heterogeneity of experimentally synthesized materials, such as defects or mixed functional groups. Second, the microkinetic simulations assume steady-state conditions and neglect potential catalyst deactivation mechanisms (e.g., coking or sintering), which are critical for real-world applications. Finally, the experimental validation of predicted activities (e.g., for V_3_N_2_O_2_) remains to be explored, as synthesis challenges or stability issues under reaction conditions could influence practical performance. Future studies combining *in situ* characterization and experimental synthesis are needed to bridge these gaps.

## Resource availability

### Lead contact

Further requests for computational data, detailed methodologies, or collaborative inquiries should be directed to the lead contact, Bo Li (boli@synu.edu.cn).

### Materials availability

This study did not generate new physical materials.

### Data and code availability

• The derivation of active site areas and rotational parameters for microkinetic modeling is available as Data S1. All other data reported in this paper will be shared by the lead contact upon request.
• This paper does not report original code.
• Any additional information required to reanalyze the data reported in this paper are available from the lead contact upon request.

## Acknowledgments

This work was supported by 10.13039/501100001809National Natural Science Foundation of China (grant nos. 22372105 and 22172100); Basic Research Project of Education Office of Liaoning Province (JYTZD2023183); the Fundamental Research Funds for the Liaoning Universities, 10.13039/501100012145Shenyang Normal University (BS202208); and the Program for Excellent Talents in Shenyang Normal University.

## Author contributions

A.A. conceived the study, developed the methodology, performed formal analysis and investigations, conducted the calculations and simulations, curated the data, wrote the original draft, and created visualizations. X.S. coordinated and supervised the research direction. Y.T. and Y.X. contributed to validation and manuscript review. B.L. provided supervision, secured project resources and funding, and participated in critical revisions of the manuscript. All authors discussed the results and contributed to the final version of the paper.

## Declaration of interests

The authors declare no competing interests.

## STAR★Methods

### Key resources table

**Table:** 

REAGENT or RESOURCE	SOURCE	IDENTIFIER
**Software and algorithms**		

VASP	VASP Software GmbH	https://www.vasp.at
VESTA software package	JP-Minerals	https://jp-minerals.org/vesta/en/
Jmol	Open-source community	https://jmol.sourceforge.net/
Bader charge analysis	Henkelman group	https://theory.cm.utexas.edu/henkelman/code/bader/
COHP	LOBSTER code	http://www.cohp.de/
p4vasp	VASP post-processing tool	http://p4vasp.at
MKMCXX program	Filot et al. (Eindhoven University)	https://wiki.mkmcxx.nl

### Method details

DFT calculations are performed by employing plane-wave-based Vienna ab initio simulation package (VASP).^36^^,^^37^ Interactions between ion cores and valence electrons are treated with projector-augmented wave (PAW) method.^38^^,^^39^ Perdew-Burke-Ernzerhof (PBE) exchange-correlation functional is selected to treat the exchange and correlation energies of electrons that are majorly applied to the studies of MXenes,^40^^,^^41^ more specifically TM carbides^42^^,^^43^ based catalytic systems. Kohn-Sham equations are solved using a plane-wave basis with an energy cutoff of 450 eV. To prevent interactions with its periodic images, a vacuum separation of 15 Å is added in z-direction of all MXenes structural models. The d-electrons of TM are treated with GGA+U scheme that is accountable for on-site Coulomb interactions^44^ and the value of U is set to be 4 eV for Ti, V, Nb, Ta and 6 eV for Mo as suggested from literature.^45^ Furthermore, Grimme’s DFT-D3 method is employed to effectively rectify van der Waals interactions.^46^ Brillouin zone is sampled with 3×3×1 Monkhorst-Pack mesh.^47^

Residual Hellmann-Feynman forces on relaxed atoms are converged to 0.05 eV.Å^-1^ during atomic structure relaxation, whilst total energy of supercell is converged to 10^-5^ eV in electronic energy optimization iterations.

The formation energy quantifies the stability of MXene relative to its isolated elemental components. The general equation for calculating the formation energy $\left({\Delta E}_{f}\right)$ is given as follows;^31^

(Equation 1) $${\Delta E}_{f}=E_{MXene}-\sum n_{i}\mu_{i}$$

where $E_{MXene}$ is the total energy of MXene (e.g. Ti_3_C_2_O_2_), $\mu_{i}$ corresponds to the chemical potential of element i (such that, Ti, C, and O), and $n_{i}$ represents the stoichiometric number of element i in the MXene.

Test calculations on a larger supercell confirmed that the adsorption energies and activation barriers remain conserved, as provided in Table S6. These results validate the reliability of our computational methodology. Adsorption energy (${\Delta E}_{ads}$) for propane molecule is calculated by the equation:

(Equation 2) $${\Delta E}_{ads}=E_{C_{3}H_{8}/MXenes}-E_{C_{3}H_{8}}-E_{MXenes}$$

where $E_{C_{3}H_{8}/MXenes}$ defines total energy of propane while interacting with the surface of MXenes, $E_{C_{3}H_{8}}$ and $E_{MXenes}$ represent individual energies of propane molecule (C_3_H_8_) and catalyst.

Bader charge analysis^48^ is carried out by integrating the electron densities per atomic basis using a code developed by Henkelman.^49^ This is further used to calculate charge densities and charge density difference between adsorbent and adsorbate. A Crystal orbital Hamilton population (COHP)^50^^,^^51^ from plane-wave DFT output is calculated by LOBSTER software.^52^

The average energy of *p*-band states ($\varepsilon_{p})$ related to terminal oxygen is given as;

(Equation 3) $$\varepsilon_{p}=\frac{\int n_{p}\left(\varepsilon \right)\varepsilon d\varepsilon }{\int n_{p}\left(\varepsilon \right)d\varepsilon }$$

wherein, $n_{p}\left(\varepsilon \right)$ represents density of states projected onto p-orbitals, while ε denotes the energy level, typically referenced to the Fermi energy.

Transition state (TS) structures are located via climbing image nudged elastic band (CI-NEB) method^53^^,^^54^ and dimer method.^55^^,^^56^ Herein, the former involves five intermediate images. Later on, frequency analysis is conducted over designated TS structures along the reaction pathway. Activation energy (E_a_) is thus computed as the difference between TS and initial state. For transition states, the imaginary frequency corresponding to the reaction coordinate is excluded from zero-point energy (ZPE) calculations.

A microkinetic analysis is devised to interpret the kinetics of PDH reaction. Identification of surface coverage of reaction intermediates and rate-determining elementary reaction of PDH is carried out using microkinetic modeling (MKM) simulations as implemented in MKMCXX program.^57^ The entropy for each adsorbed intermediate and transition state is obtained according to Equation 4. For adsorbed species (e.g., propane, propyl radicals, hydrogen), the vibrational entropy is the primary contribution, and rotation and translation are treated as frustrated vibration. The vibrational frequencies are calculated using finite displacement methods, and the entropy is evaluated using the harmonic oscillator approximation:

(Equation 4) $$S_{vib}=k_{B}\sum_{i=1}^{3N-6}\left[\frac{hv_{i}/k_{B}T}{e^{hv_{i}/k_{B}T}-1}-\ln\left(1-e^{-hv_{i}/k_{B}T}\right)\right]$$

where $v_{i}$ are the vibrational frequencies, h is Planck’s constant, and N represents the number of atoms.

For gas-phase molecules (e.g., propane, propylene, H_2_), the entropy includes translational, rotational, and vibrational contributions, which can be calculated using ideal-gas approximations implemented in MKMCXX code. The entropy terms are incorporated into the Gibbs free energy (ΔG = ΔH − TΔS) for each elementary step.

The parameters outlining the rates of surface and desorption reaction steps are approximated within the frame of transition state theory (TST).^58^^,^^59^ This incorporates activation energy barriers and partition functions accountable for vibrational degrees of freedom of ground state and TSs. Hertz-Knudsen kinetics is used to determine the reaction rates for adsorbed and desorbed species. Rate constant regarding adsorption reaction is calculated as,

(Equation 5) $$k_{ads}=\frac{PA}{\sqrt{2\pi mk_{b}T}}$$

Here, P, A, m, $k_{b}$ and T denote partial pressure of gas molecule, surface area on which molecule adsorbs, mass of reactant, Boltzmann’s constant and reaction temperature. The surface area for adsorption (A) is approximated as $1\times 10^{-19}\;m^{2}$, corresponding to the effective area per active site in a 4×4 MXene supercell, derived from DFT-optimized lattice parameters, and the details are given in Data S1.

Desorption rate constant $\left(k_{des}\right)$ can be computed by an equation;

(Equation 6) $$k_{des}=\frac{k_{b}T^{3}}{h^{3}}\;\frac{2\pi mk_{b}A}{\sigma \theta_{rot}}\;e^{\frac{-\Delta G}{k_{b}T}}$$

In this equation, h is the Planck constant, σ indicates symmetry number (σ=2 for H_2_ due to rotational symmetry and σ=1 for propylene (C_3_H_6_) due to its asymmetric structure,^60^ $\theta_{rot}$ represents the characteristic rotational temperature (85.3 K for ortho-H_2_ and 0.89 K for C_3_H_6_, calculated as the geometric mean of its principal rotational constants). The detailed derivation of these parameters is provided in Data S1. ΔG refers to change in Gibbs free energy for desorption.

The rate constants are compulsory for each elementary step including forward (k_f_) and backward (k_b_) reaction. For surface reactions, these constants can be estimated by virtue of TST using an equation;

(Equation 7) $$k=\frac{k_{B}T}{h}\;\frac{Q^{TS}}{Q}\;e^{-\frac{E_{a}}{k_{B}T}}$$

where Q and $Q^{TS}$ correspond to partition functions of initial and transition state, respectively. Both of them include vibrational modes but the latter includes vibrational modes with the exception of imaginary mode which is a characteristic of TS. Finally, $k_{B}$, h and $E_{a}$ designate Boltzmann constant, Planck constant and activation energy, accordingly.

The simulation involves time-integration of ordinary differential equations that describes the kinetics of system and the reaction is evaluated between 600 and 900 K in steps of 50 K. Steady-state coverage for each adsorbed species ($\theta_{i}$) over the surface of MXenes is estimated by solving the differential equations of each intermediate (i) with respect to time. The steady-state solution is achieved on a condition that $\frac{\partial \theta_{i}}{\partial t}$ must be zero. Afterwards, this measure of surface coverage assists in calculating the rates of individual elementary reaction steps.

To unravel the role of each individual elementary reaction step on the overall reaction rate, the degree of rate control (DRC) proposed by Campbell is used and is defined as;

(Equation 8) $$X_{RC,i}={\left[\frac{\partial lnr}{\partial \ln\;k_{i}}\right]}_{k_{j}\neq k_{i}K_{i}}$$

Here, $X_{RC,i}$ is DRC coefficient of elementary reaction step i, r denotes overall reaction rate, $k_{i}$ and $K_{i}$ refers to reaction rate constant and equilibrium constant of elementary step i, respectively.

The propylene selectivity is determined from the net production rates of all reaction products. It can be expressed as:

(Equation 9) $$S_{i}=\frac{r_{i}}{\sum_{j}r_{j}}$$

Where $S_{i}$ defines the ratio of the net production rate of propylene ($r_{i}$) to the sum of the net production rates of all relevant products ($r_{j}$).

### Quantification and statistical analysis

The computational analyses in this study were performed using several specialized software packages. Density functional theory (DFT) calculations were performed using the Vienna Ab Initio Simulation Package (VASP), implementing strict convergence criteria of 10^-5^ eV for energy and 0.05 eV/Å for forces (detailed in method details and Table S6). VASP facilitated structural optimizations, electronic structure analyses, and transition state searches through CI-NEB and dimer methods. Structural visualization and charge density distributions were generated using VESTA and Jmol (Figures 1, 2, S1, S2, and S11). Electronic properties were quantified through Bader charge analysis (Figure 3A; Table S1) and Crystal Orbital Hamilton Population (COHP) calculations using the LOBSTER code (Figure 3B). Post-processing of electronic structure data utilized p4vasp (Figure S5). Microkinetic modeling, implemented via the MKMCXX program, provided turnover frequencies and reaction rates (Figures 8, 9, and S13–S16), with rate control analysis revealing key mechanistic insights (Figures S18 and S19). The reaction network was visualized using Graphviz (Figure S17). The study evaluated a total of 20 independent catalyst configurations, comprising combinations of five transition metals (Ti, V, Nb, Mo, Ta) with four distinct X-layer substitutions (TM-C, TM-N, TM-C/N, TM-N/C). Each configuration was analyzed systematically to assess its catalytic performance. Complete methodological details, software specifications, and all statistical parameters are documented in the method details section and Supplemental Information (Figures S1–S19; Tables S1–S6).

### Additional resources

All data generated during this computational study are included in the manuscript and supplementary information. No external resources were required.

## Supplemental information

Document S1. Figures S1–S19 and Tables S2–S6

Data S1. Derivation of active site area and rotational parameters for microkinetic modeling, related to STAR Methods

Table S1. Atomic coordinates for representative MXene models Atomic positions (x, y, z) for Ti_3_C_2_O_2_ (TM-C), Ti_3_N_2_O_2_ (TM-N), Ti_3_(CN)O_2_ (TM-C/N mixed layer), and Ti_3_(NC)O_2_ (TM-N/C mixed layer), derived from DFT-optimized structures, related to Figure 1.

## References

[1] Loiland J.A., Zhao Z., Patel A., Hazin P. (2019). Boron-containing catalysts for the oxidative dehydrogenation of ethane/propane mixtures. Ind. Eng. Chem. Res..

[2] Sattler J.J.H.B., Ruiz-Martinez J., Santillan-Jimenez E., Weckhuysen B.M. (2014). Catalytic dehydrogenation of light alkanes on metals and metal oxides. Chem. Rev..

[3] Chen S., Chang X., Sun G., Zhang T., Xu Y., Wang Y., Pei C., Gong J. (2021). Propane dehydrogenation: catalyst development, new chemistry, and emerging technologies. Chem. Soc. Rev..

[4] Nykanen L., Honkala K. (2013). Selectivity in propene dehydrogenation on Pt and Pt_3_Sn surfaces from first principles. ACS Catal..

[5] Michorczyk P., Ogonowski J., Kus'trowski P., Chmielarz L. (2008). Chromium oxide supported on MCM-41 as a highly active and selective catalyst for dehydrogenation of propane with CO_2_. Appl. Catal. A Gen..

[6] Wang G., Zhu X., Li C. (2020). Recent progress in commercial and novel catalysts for catalytic dehydrogenation of light alkanes. Chem. Rec..

[7] Abedin M.A., Kanitkar S., Bhattar S., Spivey J.J. (2020). Mo oxide supported on sulfated hafnia: Novel solid acid catalyst for direct activation of ethane & propane. Appl. Catal. A Gen..

[8] Yang M.L., Zhu Y.A., Fan C., Sui Z.J., Chen D., Zhou X.G. (2011). DFT study of propane dehydrogenation on Pt catalyst: effects of step sites. Phys. Chem. Chem. Phys..

[9] Lian Z., Ali S., Liu T., Si C., Li B., Su D.S. (2018). Revealing the janus character of the coke precursor in the propane direct dehydrogenation on Pt catalysts from a kMC simulation. ACS Catal..

[10] Huš M., Kopač D., Likozar B. (2020). Kinetics of non-oxidative propane dehydrogenation on Cr_2_O_3_ and the nature of catalyst deactivation from first-principles simulations. J. Catal..

[11] Michorczyk P., Pietrzyk P., Ogonowski J. (2012). Preparation and characterization of SBA-1-supported chromium oxide catalysts for CO_2_ assisted dehydrogenation of propane. Microporous Mesoporous Mater..

[12] Ascoop I., Galvita V.V., Alexopoulos K., Reyniers M.F., Van Der Voort P., Bliznuk V., Marin G.B. (2016). The role of CO_2_ in the dehydrogenation of propane over WO-VO/SiO_2_. J. Catal..

[13] Zhang J., Zhou R.J., Chang Q.Y., Sui Z.J., Zhou X.G., Chen D., Zhu Y.A. (2021). Tailoring catalytic properties of V_2_O_3_ to propane dehydrogenation through single-atom doping: A DFT study. Catal. Today.

[14] Carrero C.A., Schlögl R., Wachs I.E., Schomaecker R. (2014). Critical Literature Review of the Kinetics for the Oxidative Dehydrogenation of Propane over Well-Defined Supported Vanadium Oxide Catalysts. ACS Catal..

[15] Lawson S., Newport K.A., Axtell A., Boucher C., Grant B., Haas M., Lee M., Rezaei F., Rownaghi A.A. (2021). Structured bifunctional catalysts for CO_2_ activation and oxidative dehydrogenation of propane. ACS Sustain. Chem. Eng..

[16] Naguib M., Kurtoglu M., Presser V., Lu J., Niu J., Heon M., Hultman L., Gogotsi Y., Barsoum M.W. (2011). Two-dimensional nanocrystals: two-dimensional nanocrystals produced by exfoliation of Ti_3_AlC_2_ (Adv. Mater. 37/2011). Adv. Mater..

[17] Magne D., Mauchamp V., Célérier S., Chartier P., Cabioc'h T. (2016). Site-projected Electronic Structure of Two-dimensional Ti_3_C_2_ MXene: the Role of the Surface Functionalization Groups. Phys. Chem. Chem. Phys..

[18] Li Z., Wu Y. (2019). 2D early transition metal carbides (MXenes) for catalysis. Small.

[19] Ng W.H.K., Gnanakumar E.S., Batyrev E., Sharma S.K., Pujari P.K., Greer H.F., Zhou W., Sakidja R., Rothenberg G., Barsoum M.W., Shiju N.R. (2018). The Ti_3_AlC_2_ MAX phase as an efficient catalyst for oxidative dehydrogenation of n-butane. Angew. Chem..

[20] Diao J., Hu M., Lian Z., Li Z., Zhang H., Huang F., Li B., Wang X., Su D.S., Liu H. (2018). Ti_3_C_2_T_x_ MXene catalyzed ethylbenzene dehydrogenation: active sites and mechanism exploration from both experimental and theoretical aspects. ACS Catal..

[21] Niu K., Chi L., Rosen J., Björk J. (2020). C-H activation of light alkanes on MXenes predicted by hydrogen affinity. Phys. Chem. Chem. Phys..

[22] Niu K., Chi L., Rosen J., Björk J. (2021). Structure-activity correlation of Ti_2_CT_2_ MXenes for C-H activation. J. Phys. Condens. Matter.

[23] Sarycheva A., Gogotsi Y. (2020). Raman spectroscopy analysis of the structure and surface chemistry of Ti_3_C_2_T_x_ MXene. Chem. Mater..

[24] Ghidiu M., Lukatskaya M.R., Zhao M.Q., Gogotsi Y., Barsoum M.W. (2014). Conductive two-dimensional titanium carbide “clay” with high volumetric capacitance. Nature.

[25] Naguib M., Mashtalir O., Carle J., Presser V., Lu J., Hultman L., Gogotsi Y., Barsoum M.W. (2012). Two-dimensional transition metal carbides. ACS Nano.

[26] Naguib M., Unocic R.R., Armstrong B.L., Nanda J. (2015). Large-scale delamination of multi-layers transition metal carbides and carbonitrides “MXenes”. Dalton Trans..

[27] Soundiraraju B., George B.K. (2017). Two-Dimensional Titanium Nitride (Ti_2_N) MXene: Synthesis, Characterization, and Potential Application as Surface-Enhanced Raman Scattering Substrate. ACS Nano.

[28] Djire A., Bos A., Liu J., Zhang H., Miller E.M., Neale N.R. (2019). Pseudocapacitive Storage in Nanolayered Ti_2_NT_x_ MXene Using Mg-Ion Electrolyte. ACS Appl. Nano Mater..

[29] Venkateshalu S., Cherusseri J., Karnan M., Kumar K.S., Kollu P., Sathish M., Thomas J., Jeong S.K., Grace A.N. (2020). New method for the synthesis of 2D vanadium nitride (MXene) and its application as a supercapacitor electrode. ACS Omega.

[30] Naguib M., Mochalin V.N., Barsoum M.W., Gogotsi Y. (2014). 25th anniversary article: MXenes: a new family of two-dimensional materials. Adv. Mater..

[31] Pandey M., Thygesen K.S. (2017). Two-dimensional MXenes as catalysts for electrochemical hydrogen evolution: A computational screening study. J. Phys. Chem. C.

[32] Mishra A., Srivastava P., Carreras A., Tanaka I., Mizuseki H., Lee K.R., Singh A.K. (2017). Atomistic origin of phase stability in oxygen-functionalized MXene: A comparative study. J. Phys. Chem. C.

[33] Gu Y., Liu H., Yang M., Ma Z., Zhao L., Xing W., Wu P., Liu X., Mintova S., Bai P., Yan Z. (2020). Highly stable phosphine modified VO_x_/Al_2_O_3_ catalyst in propane dehydrogenation. Appl. Catal. B Environ..

[34] Zhu J., Yang M.L., Yu Y., Zhu Y.A., Sui Z.J., Zhou X.G., Holmen A., Chen D. (2015). Size-dependent reaction mechanism and kinetics for propane dehydrogenation over Pt catalysts. ACS Catal..

[35] Li B., Su D. (2014). The nucleophilicity of the oxygen functional groups on carbon materials: a DFT analysis. Chem. Eur J..

[36] Kresse G., Hafner J. (1993). Ab initio molecular dynamics for liquid metals. Phys. Rev. B.

[37] Kresse G., Furthmüller J. (1996). Efficient iterative schemes for ab initio total-energy calculations using a plane-wave basis set. Phys. Rev. B.

[38] Blöchl P.E. (1994). Projector augmented-wave method. Phys. Rev. B.

[39] Kresse G., Joubert D. (1999). From ultrasoft pseudopotentials to the projects augmented-wave method. Phys. Rev. B.

[40] Keyhanian M., Farmanzadeh D., Morales-García Á., Illas F. (2022). Effect of oxygen termination on the interaction of first row transition metals with M_2_C MXenes and the feasibility of single-atom catalysts. J. Mater. Chem. A.

[41] Gouveia J.D., Morales-García Á., Vines F., Illas F., Gomes J.R. (2020). MXenes as promising catalysts for water dissociation. Appl. Catal. B Environ..

[42] Jimenez-Orozco C., Flórez E., Viñes F., Rodriguez J.A., Illas F. (2020). Critical hydrogen coverage effect on the hydrogenation of ethylene catalyzed by δ-MoC (001): An ab initio thermodynamic and kinetic study. ACS Catal..

[43] Li J., Sun L., Wan Q., Lin J., Lin S., Wang X. (2021). α-MoC supported noble metal catalysts for water-gas shift reaction: Single-atom promoter or single-atom player. J. Phys. Chem. Lett..

[44] Dudarev S.L., Botton G.A., Savrasov S.Y., Humphreys C.J., Sutton A.P. (1998). Electron-energy-loss spectra and the structural stability of nickel oxide: An LSDA+U study. Phys. Rev. B.

[45] Anasori B., Xie Y., Beidaghi M., Lu J., Hosler B.C., Hultman L., Kent P.R.C., Gogotsi Y., Barsoum M.W. (2015). Two-dimensional, ordered, double transition metals carbides (MXenes). ACS Nano.

[46] Grimme S. (2006). Semiempirical GGA-type density functional constructed with a langrange dispersion correction. J. Comput. Chem..

[47] Monkhorst H.J., Pack J.D. (1976). Special points for Brillouin zone integrations. Phys. Rev. B.

[48] Bader R.F.W. (1990).

[49] Henkelman G., Arnaldsson A., Jónsson H. (2006). A fast and robust algorithm for Bader decomposition of charge density. Comput. Mater. Sci..

[50] Dronskowski R., Blöchl P.E. (1993). Crystal orbital Hamilton populations (COHP): Energy-resolved visualization of chemical bonding in solids based on density functional calculations. J. Phys. Chem..

[51] Deringer V.L., Tchougréeff A.L., Dronskowski R. (2011). Crystal orbital Hamilton population (COHP) analysis as projected from plane-wave basis sets. J. Phys. Chem. A.

[52] Maintz S., Deringer V.L., Tchougréeff A.L., Dronskowski R. (2016). LOBSTER: A tool to extract chemical bonding from plane-wave based DFT. J. Comput. Chem..

[53] Henkelman G., Jónsson H. (2000). Improved tangent estimate in the nudged elastic band method for finding minimum energy paths and saddle points. J. Chem. Phys..

[54] Henkelman G., Uberuaga B.P., Jónsson H. (2000). A climbing image nudged elastic band method for finding saddle points and minimum energy paths. J. Chem. Phys..

[55] Henkelman G., Jónsson H. (1999). A dimer method for finding saddle points on high dimensional potential surfaces using only first derivatives. J. Chem. Phys..

[56] Heyden A., Bell A.T., Keil F.J. (2005). Efficient methods for finding transition states in chemical reactions: Comparison of improved dimer method and partitioned rational function optimization method. J. Chem. Phys..

[57] Filot I.A.W., Van Santen R.A., Hensen E.J.M. (2014). The optimally performing Fischer-Tropsch catalyst. Angew. Chem. Int. Ed..

[58] Laldler K.J., King M.C. (1983). The development of transition-state theory. J. Chem. Phys..

[59] Truhlar D.G., Garrett B.C., Klippenstein S.J. (1996). Current status of transition-state theory. J. Phys. Chem..

[60] Domalski E.S., Hearing E.D. (1988). Estimation of the Thermodynamic Properties of Hydrocarbons at 298.15 K. J. Phys. Chem. Ref. Data.

