# Supplementary material for: Tailoring the catalytic performance of MXenes in propane dehydrogenation by layer substitution from microkinetic simulations
Source: iScience. 2025 Sep 7;28(10):113480. doi: 10.1016/j.isci.2025.113480 (PMC12483649; doi:10.1016/j.isci.2025.113480)
Supplement: Document S1. Figures S1–S19 and Tables S2–S6 [file mmc1.pdf]

iScience, Volume *28*

Supplemental information

Tailoring the catalytic performance of MXenes in propane dehydrogenation by layer substitution from microkinetic simulations

Aqsa Abid, XiaoYing Sun, Yuqing Tang, Yi Xiao, and Bo Li

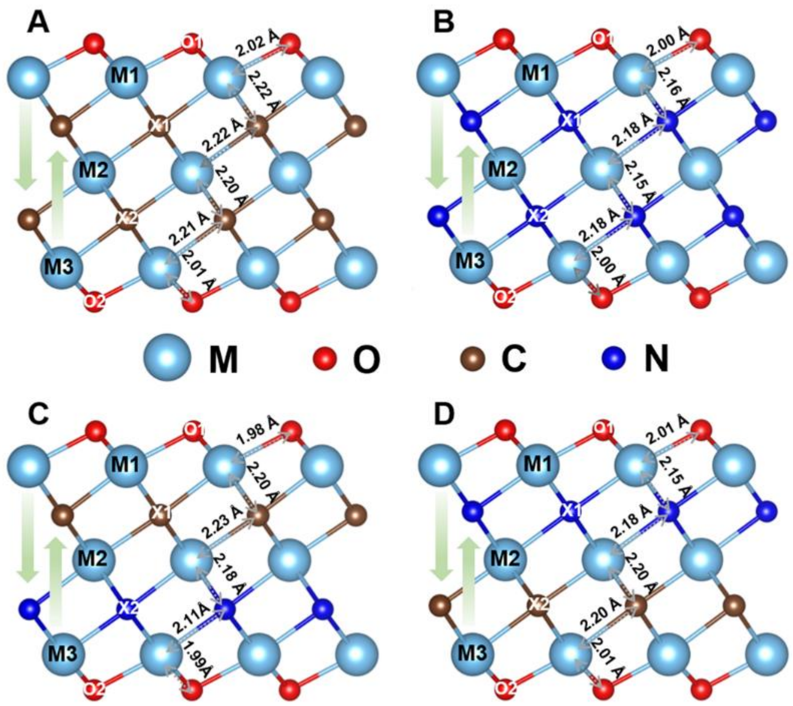

Figure S1. Optimized structures and bond lengths. Side view of optimized structure of (A) TM-C, (B) TM-N, (C) TM-C/N, and (D) TM-N/C. Bond-lengths are shown for comparison. Up and down arrows indicate front and back atoms, respectively, related to Figure 1.

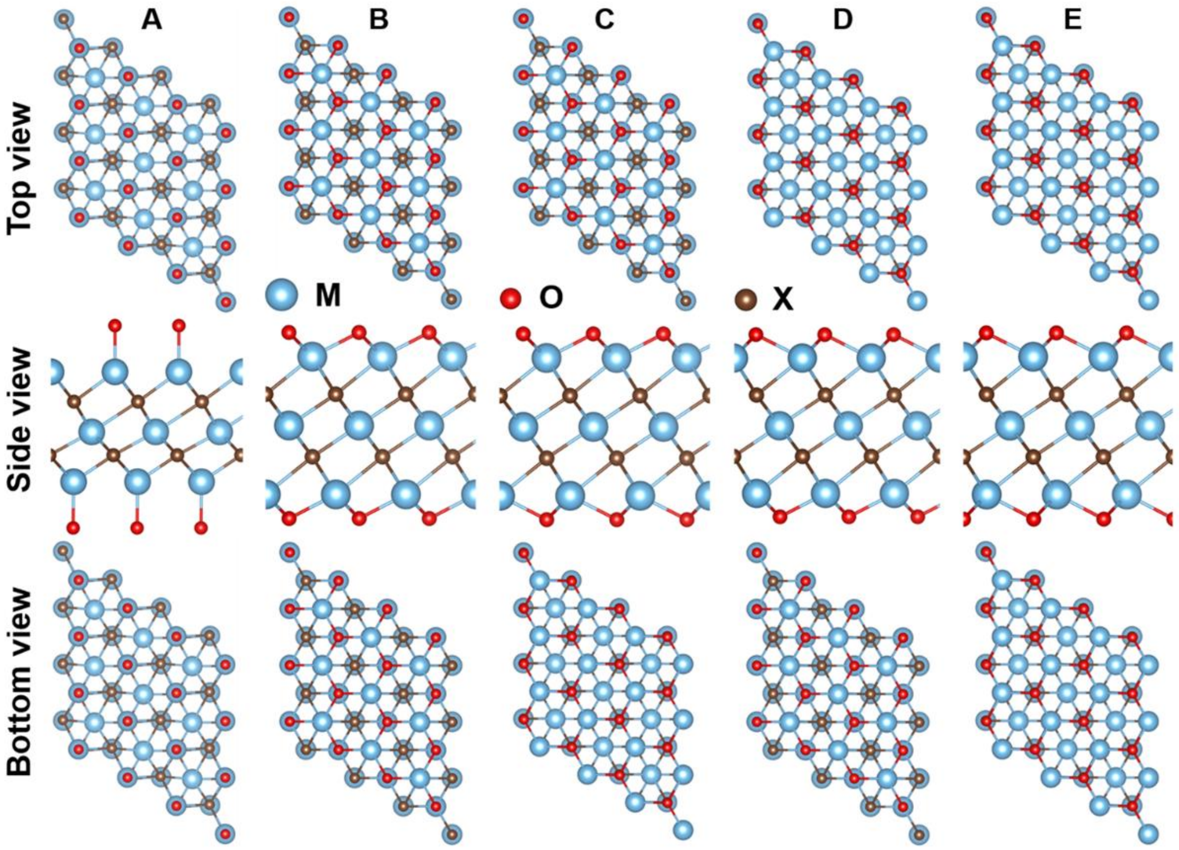

Figure S2. The top, side and bottom view of five possible oxygen functionalization sites on double layer MXenes ($M_3X_2O_2$). (A) top-top, (B) fcc-fcc, (C) fcc-hcp, (D) hcp-fcp, and (E) hcp-hcp sites. The first and second labels denote the site location on the upper and lower surfaces along the c-axis, respectively, related to Figure 1.

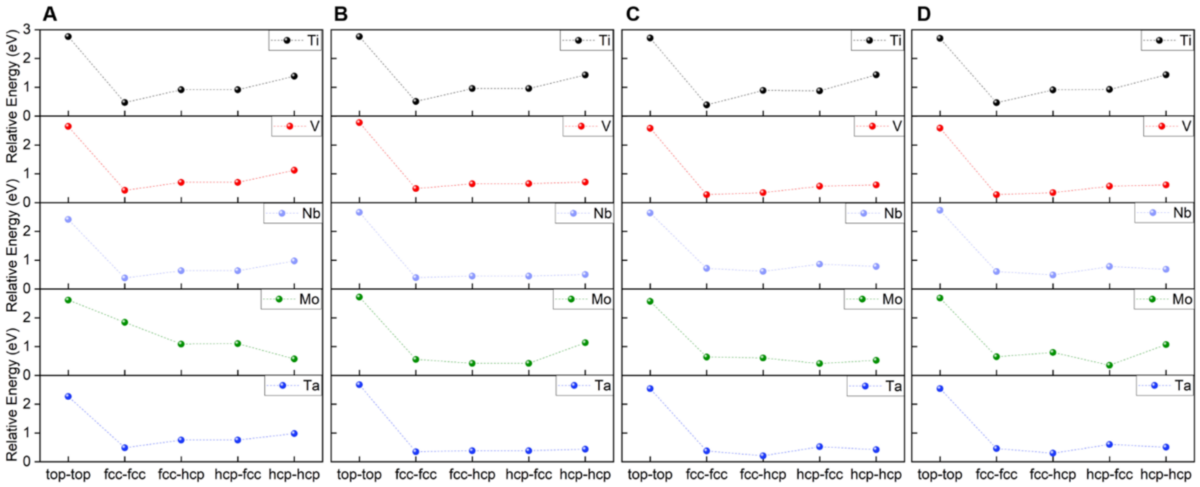

Figure S3. Relative Energy profiles for the unit cell of each MXene type with different oxygen configurations. (A) TM-C, (B) TM-N, (C) TM-C/N, and (D) TM-N/C, related to Figure 1.

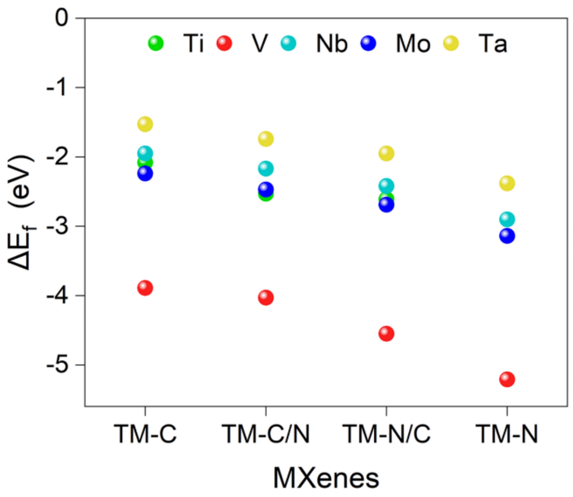

Figure S4. Calculated formation energies (ΔE_f) for double-layer MXenes, including; TM-C, TM-N, TM-C/N, TM-N/C (TM = Ti, V, Nb, Mo, and Ta), related to Figures 1 and 2.

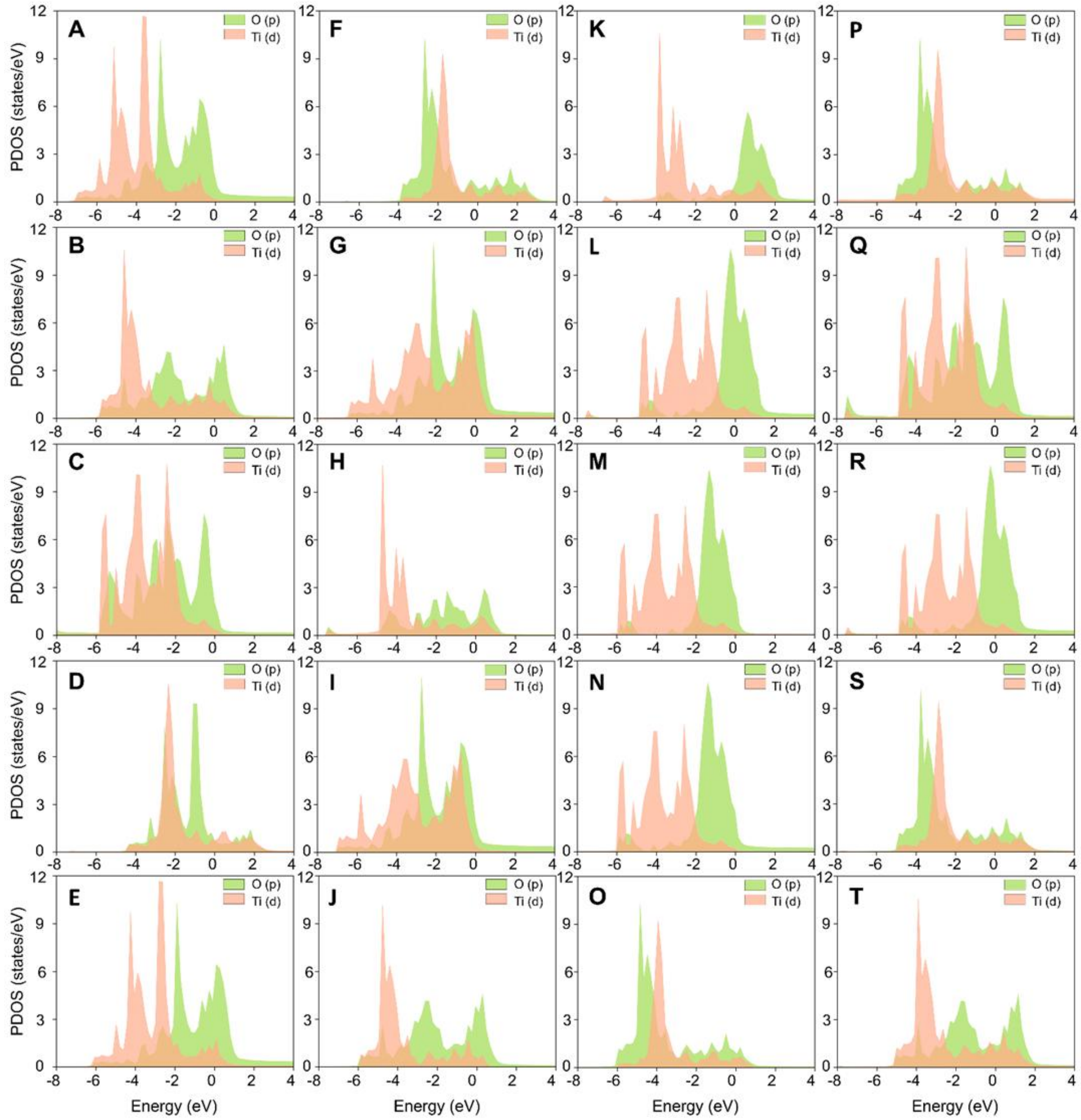

Figure S5. Illustrating projected density of states (PDOS) associated with p-orbital of terminal O atoms (green) and d-orbital of sub-layer metal atoms (orange). (A-E) TM-C, (F-J) TM-N, (K-O) TM-C/N, and (P-T) TM-N/C. Here, TM represents Ti, V, Nb, Mo, and Ta, in sequence, related to Figure 3.

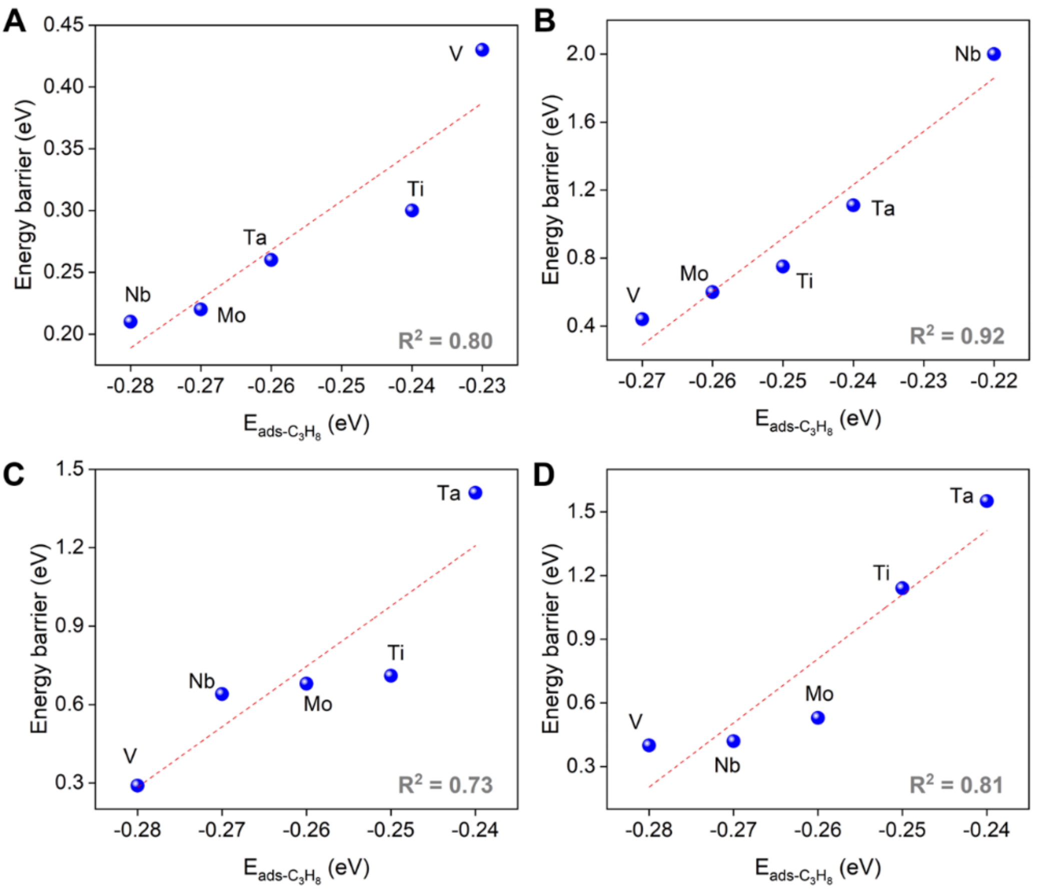

Figure S6. Correlation between propane adsorption energy and first C–H activation energy. (A) TM-C, (B) TM-N, (C) TM-C/N, and (D) TM-N/C, related to Figure 4.

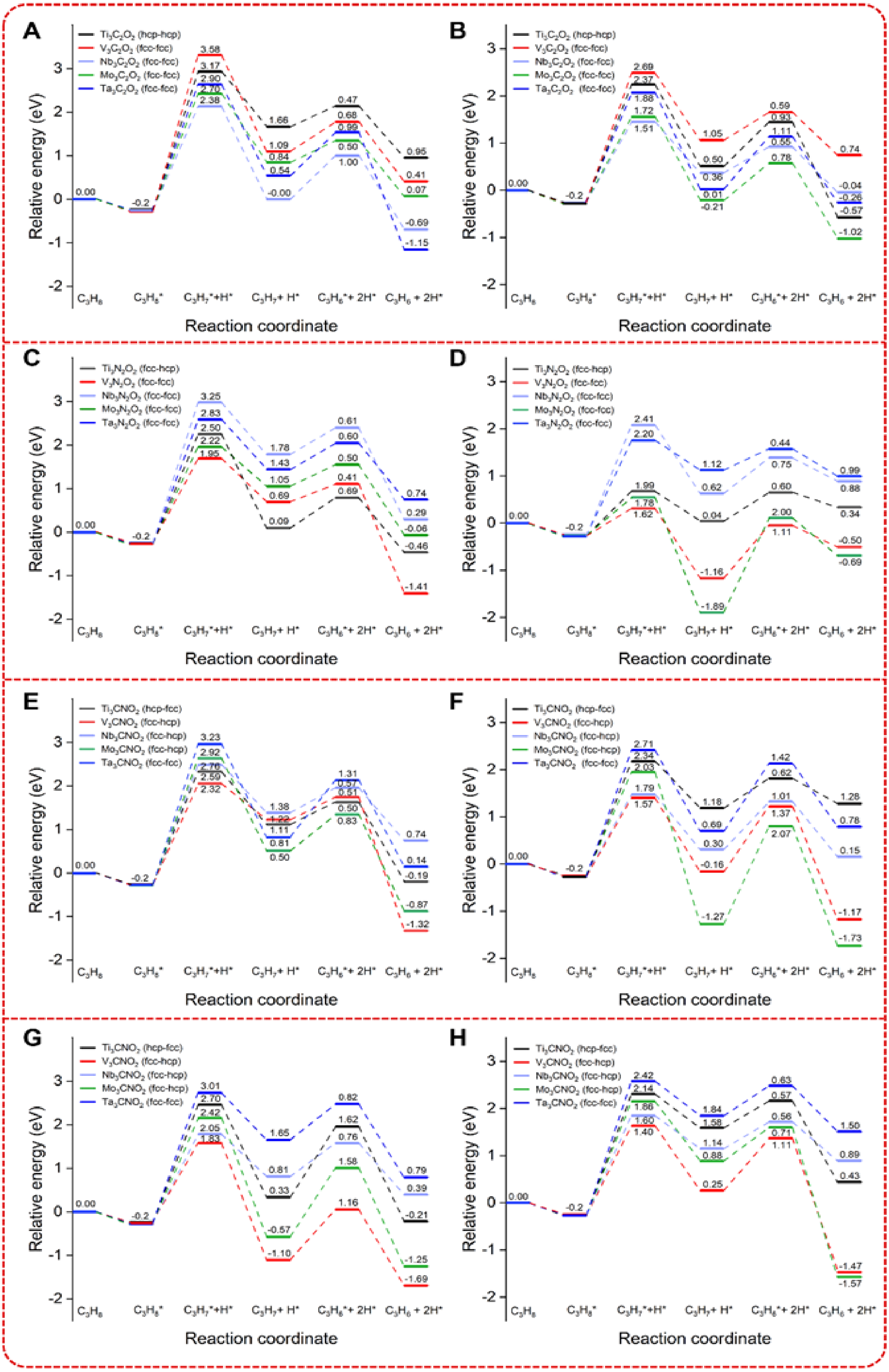

Figure S7. Potential energy profile of PDH reaction on R1 pathway. (A, B) TM-C, (C, D) TM-N, (E, F) TM-C/N, and (G, H) TM-N/C via R1-M1 (left) and R1-M2 (right), related to Figure 4.

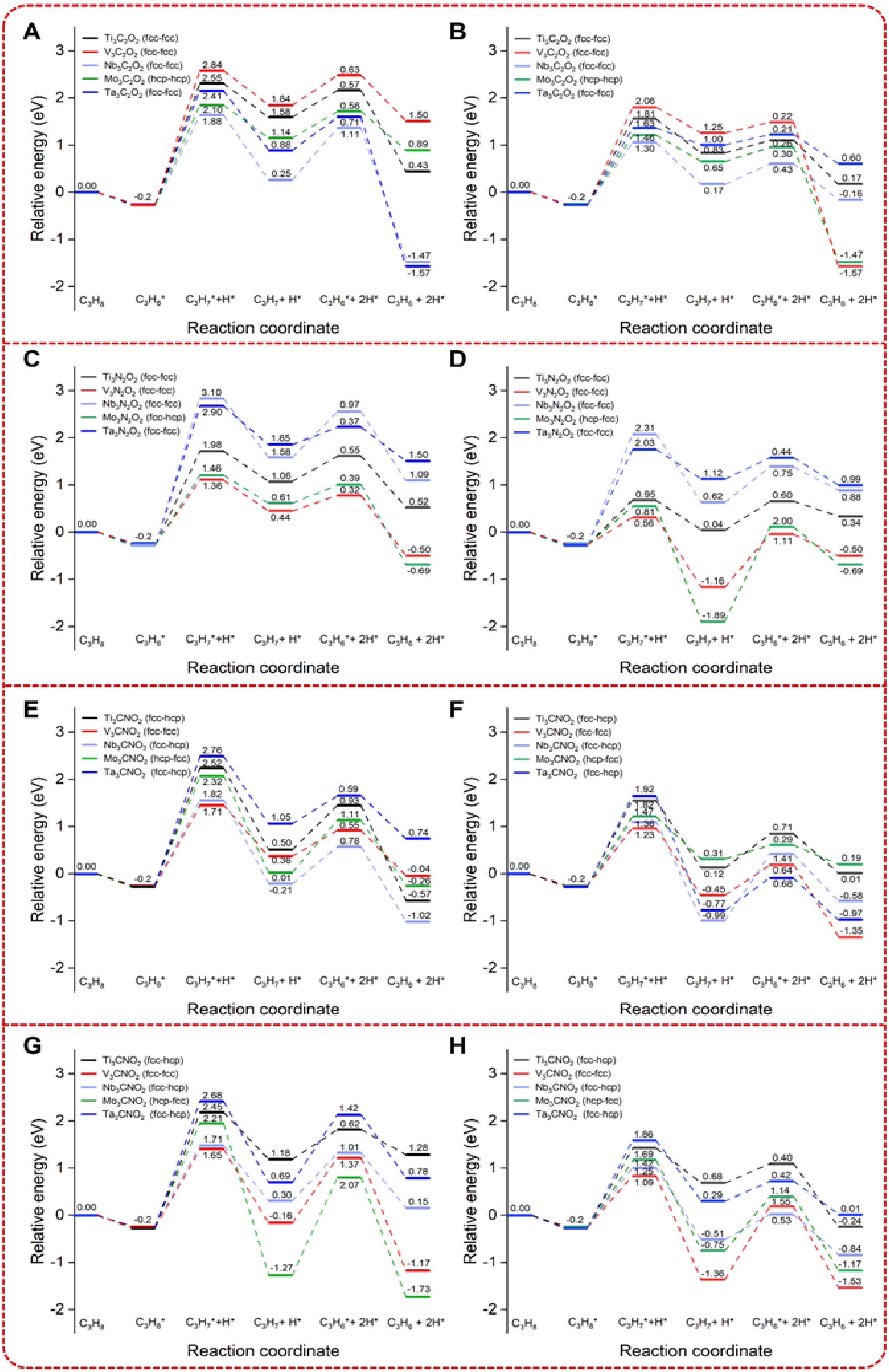

Figure S8. Potential energy profile of PDH reaction on R2 pathway. (A, B) TM-C, (C, D) TM-N, (E, F) TM-C/N, and (G, H) TM-N/C via R2-M1 (left) and R2-M2 (right), related to Figure 4.

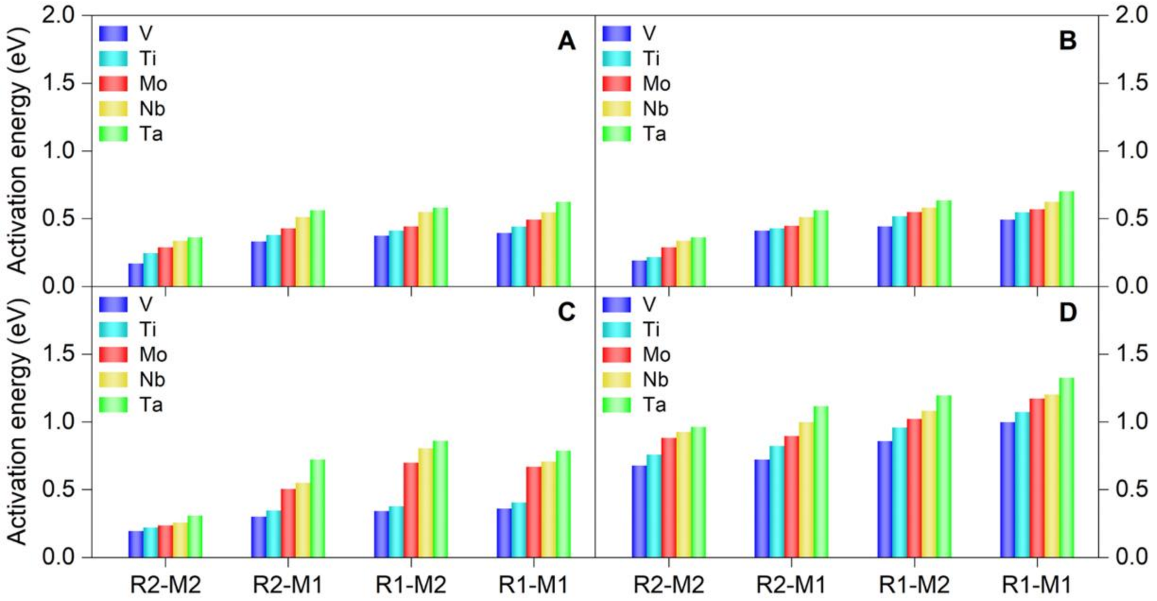

Figure S9. The activation energy for cleaving the second C-H bond of propane. Energies are shown for the reaction pathways R2-M2, R2-M1, R1-M2, and R1-M1 (outlined in Scheme 1) on (A) TM-N, (B) TM-N/C, (C) TM-C/N, and (D) TM-C, related to Figure 4.

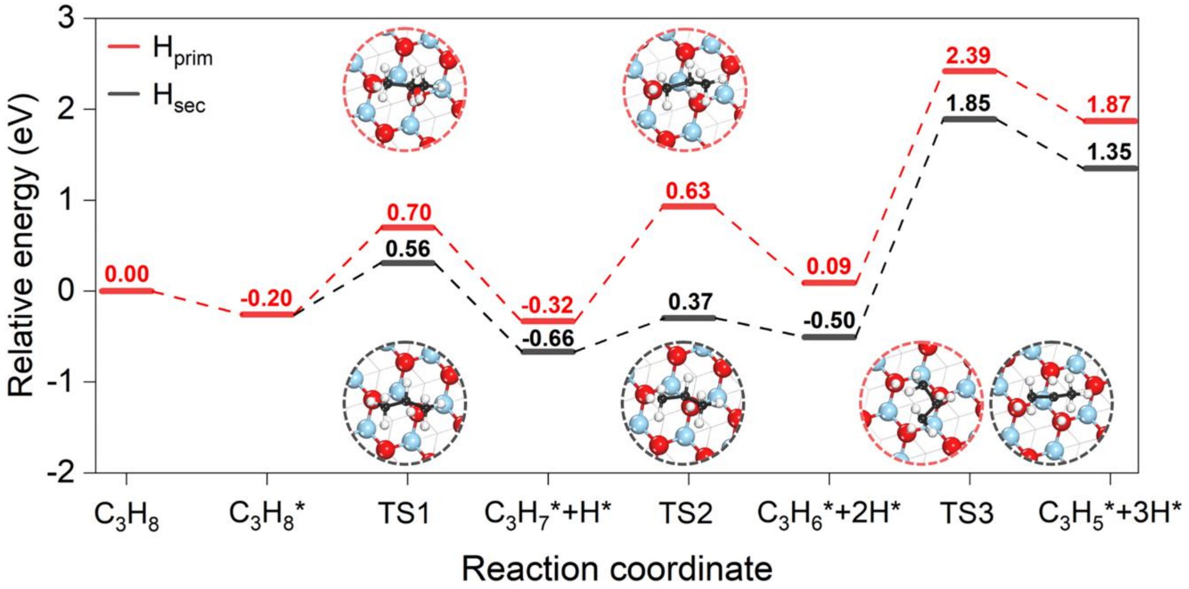

Figure S10. The potential energy profile of the most promising $V_3N_2O_2$ catalyst involving deep dehydrogenation steps during PDH reaction, related to Figure 6.

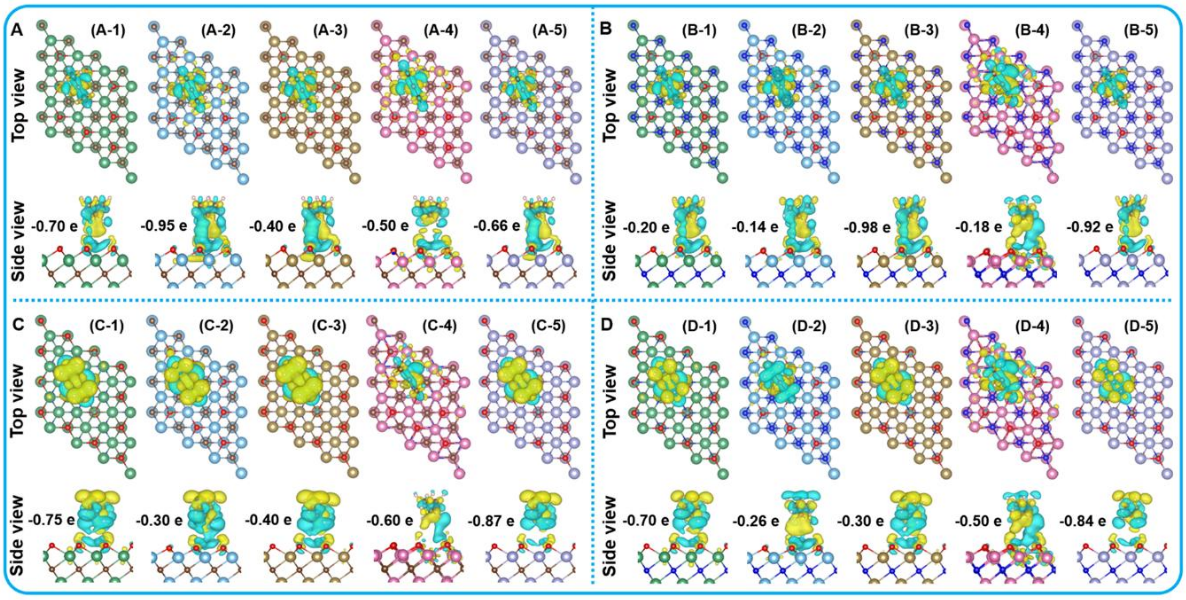

Figure S11. Charge density difference analysis of propane adsorption on MXenes. (A) TM-C, (B) TM-N, (C) TM-C/N, and (D) TM-N/C wherein, 1-5 indicates Ti, V, Nb, Mo, and Ta, respectively. Isosurface value is set to 3.5×10^{-5} e.Å^{-3}. Yellow and blue regions represent electron accumulation and depletion, respectively, related to Figure 4.

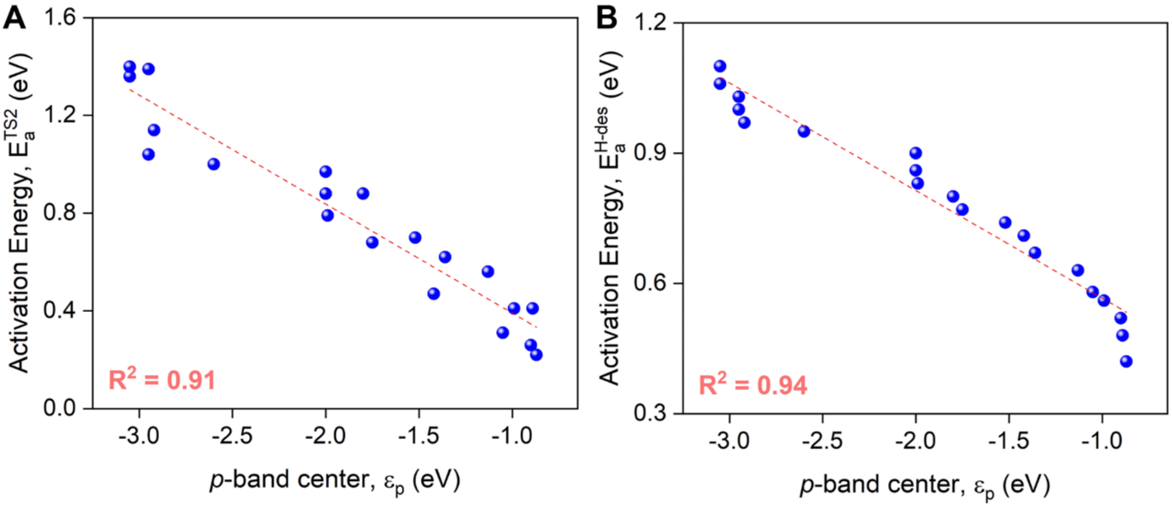

Figure S12. Scaling relations between the oxygen p-band center (ε_p) and activation barriers for propane dehydrogenation on MXenes. Correlation with (A) second activation energy barrier (E_a^{TS2}), and (B) hydrogen desorption barrier (E_a^{H-des}), related to Figure 7.

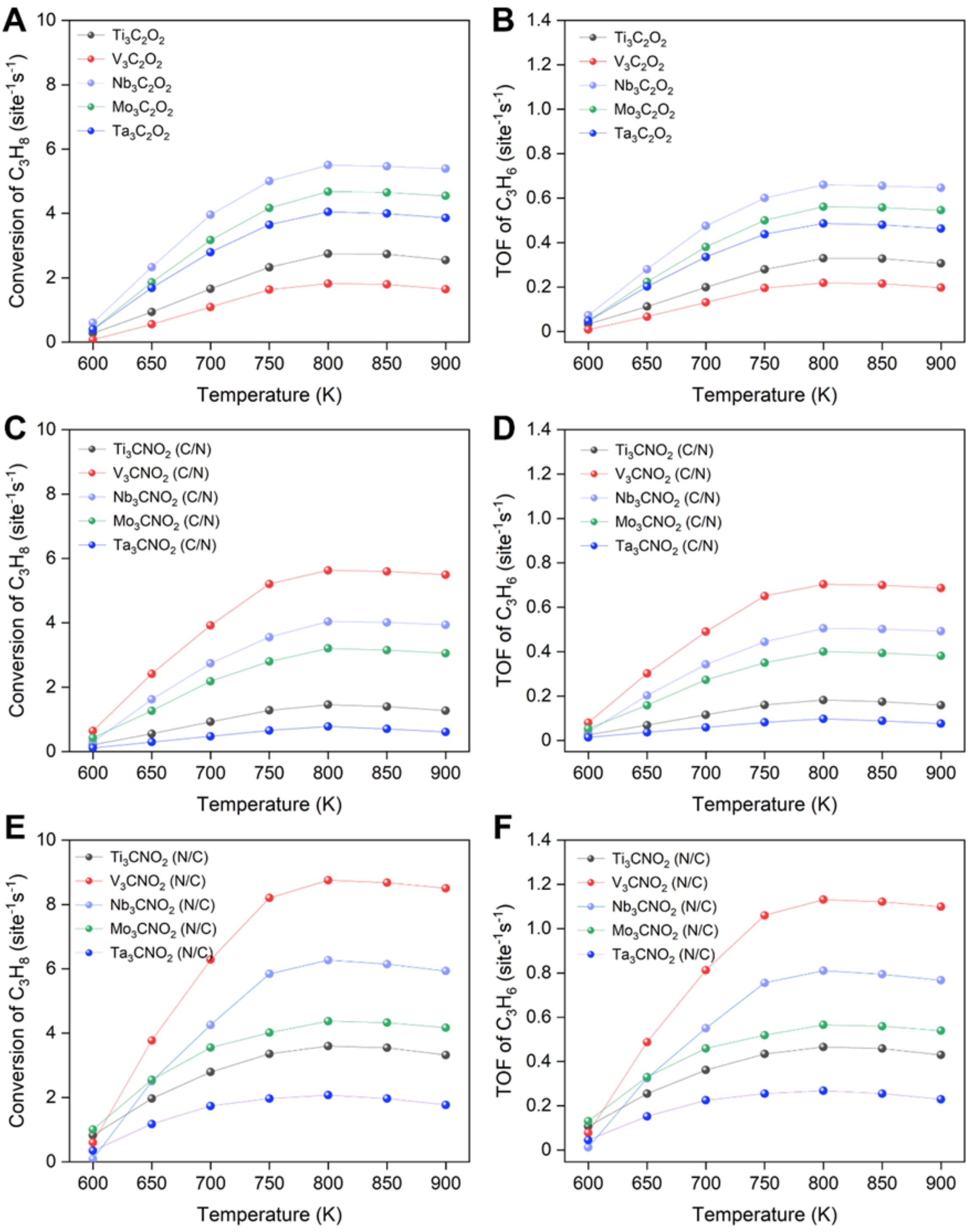

Figure S13. Propane conversion (left panels) and TOF of propylene formation (right panels) as a function of temperature (600–900 K). (A, B) TM-C, (C, D) TM-C/N, and (E, F) TM-N/C, related to Figure 8.

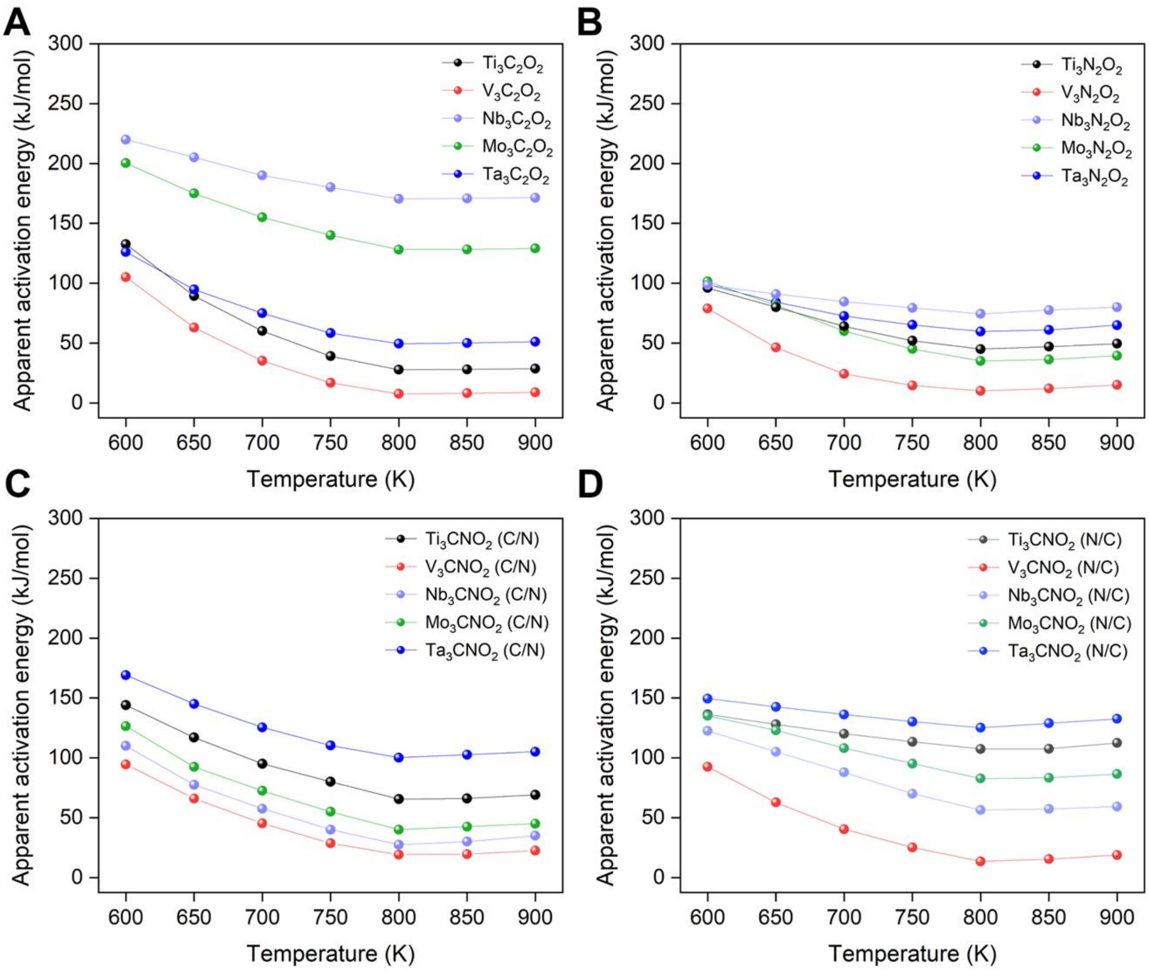

Figure S14. Apparent activation energy as a function of temperature. (A) TM-C, (B) TM-N, (C) TM-C/N, and (D) TM-N/C, related to Figure 8.

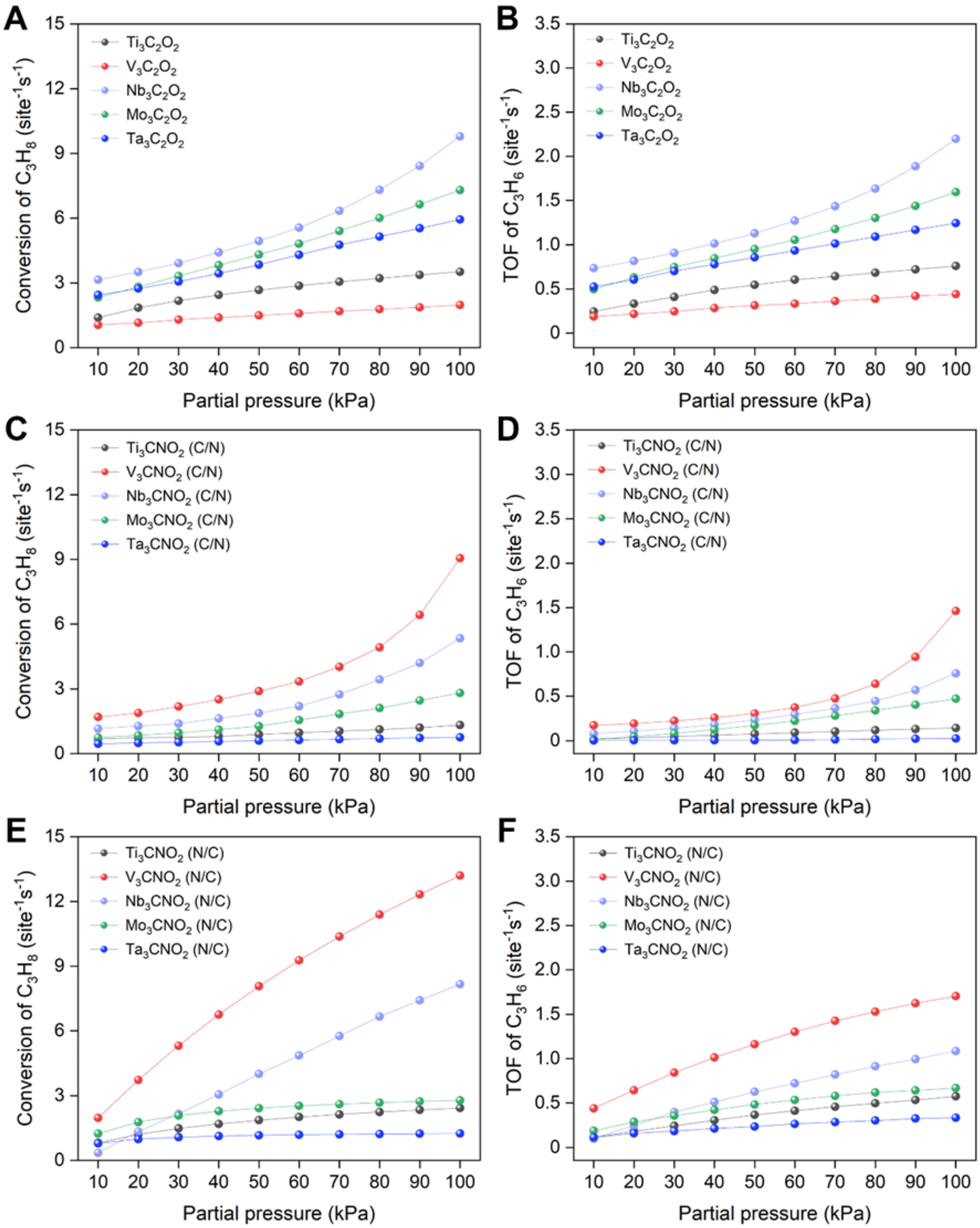

Figure S15. Propane conversion and propylene TOF as a function of propane partial pressure (kPa) at 800 K. (A, B) TM-C, (C, D) TM-C/N, and (E, F) TM-N/C, related to Figure 8.

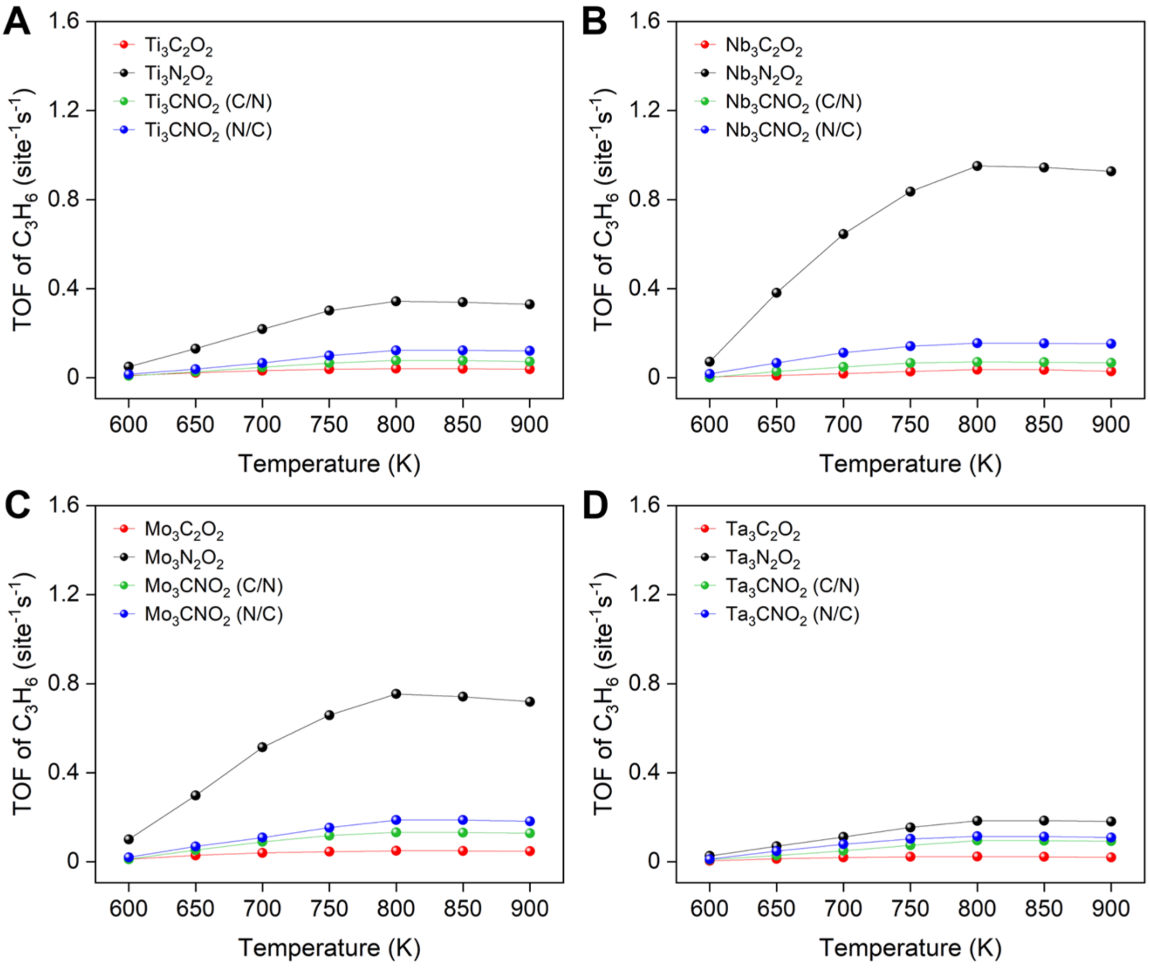

Figure S16. A comparison of propylene TOF for each substituted layer in MXenes. (A) $Ti_3X_2O_2$, (B) $Nb_3X_2O_2$, (C) $Mo_3X_2O_2$, and (D) $Ta_3X_2O_2$, related to Figure 9.

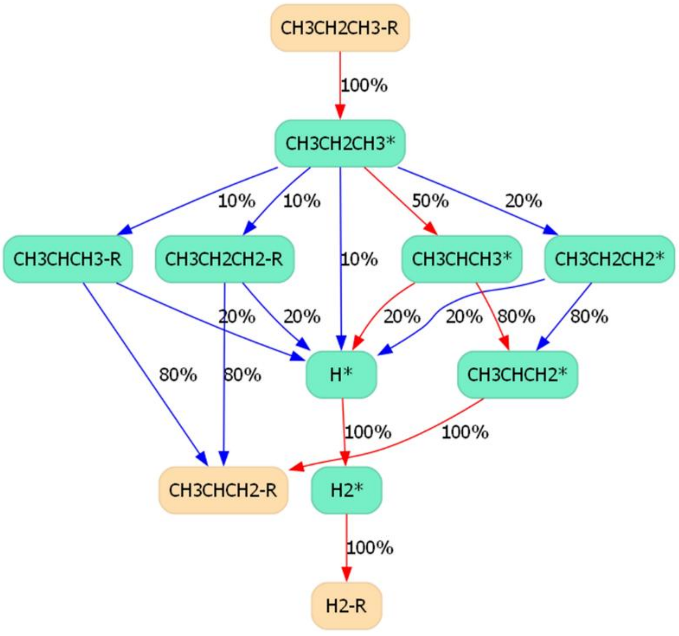

Figure S17. The reactant flux analysis of $V_3N_2O_2$ based on conversion rate of reaction species (shown in percentage). The reaction pathways including R1-M1 (1-C_3H_7), R1-M2 (1-C_3H_7*), R2-M1 (2-C_3H_7), and R2-M2 (2-C_3H_7*), in which reactant and products are represented by brown, and intermediate species are shown by green. Red arrows represent main reaction pathway, related to Scheme 1.

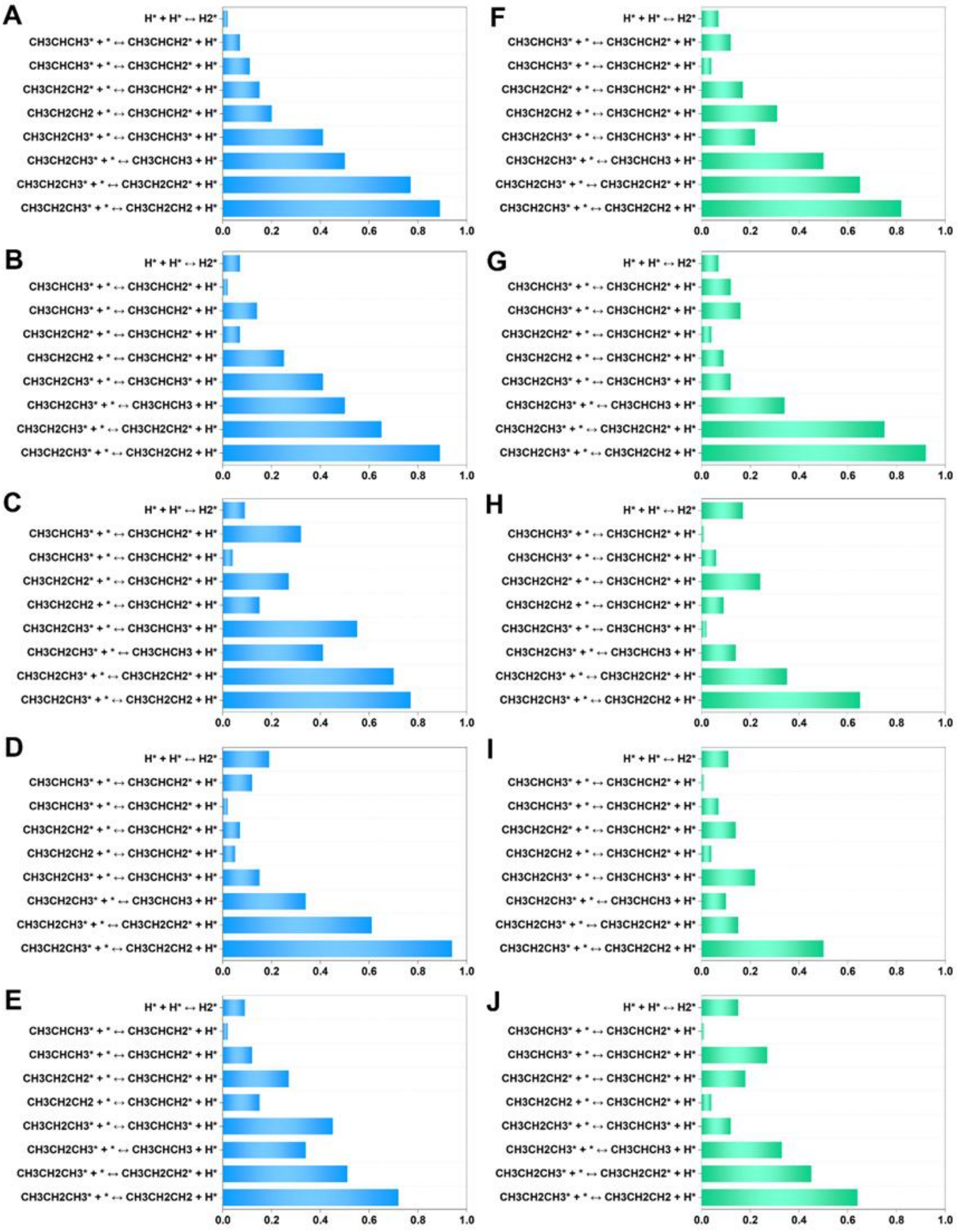

Figure S18. DRC coefficients (X_{RC}) for Arrhenius type elementary reactions in PDH at 800 K and 1 bar. (A-E) TM-C, (F-J) TM-N, where TM = Ti, V, Nb, Mo, Ta, respectively, related to Figure 9.

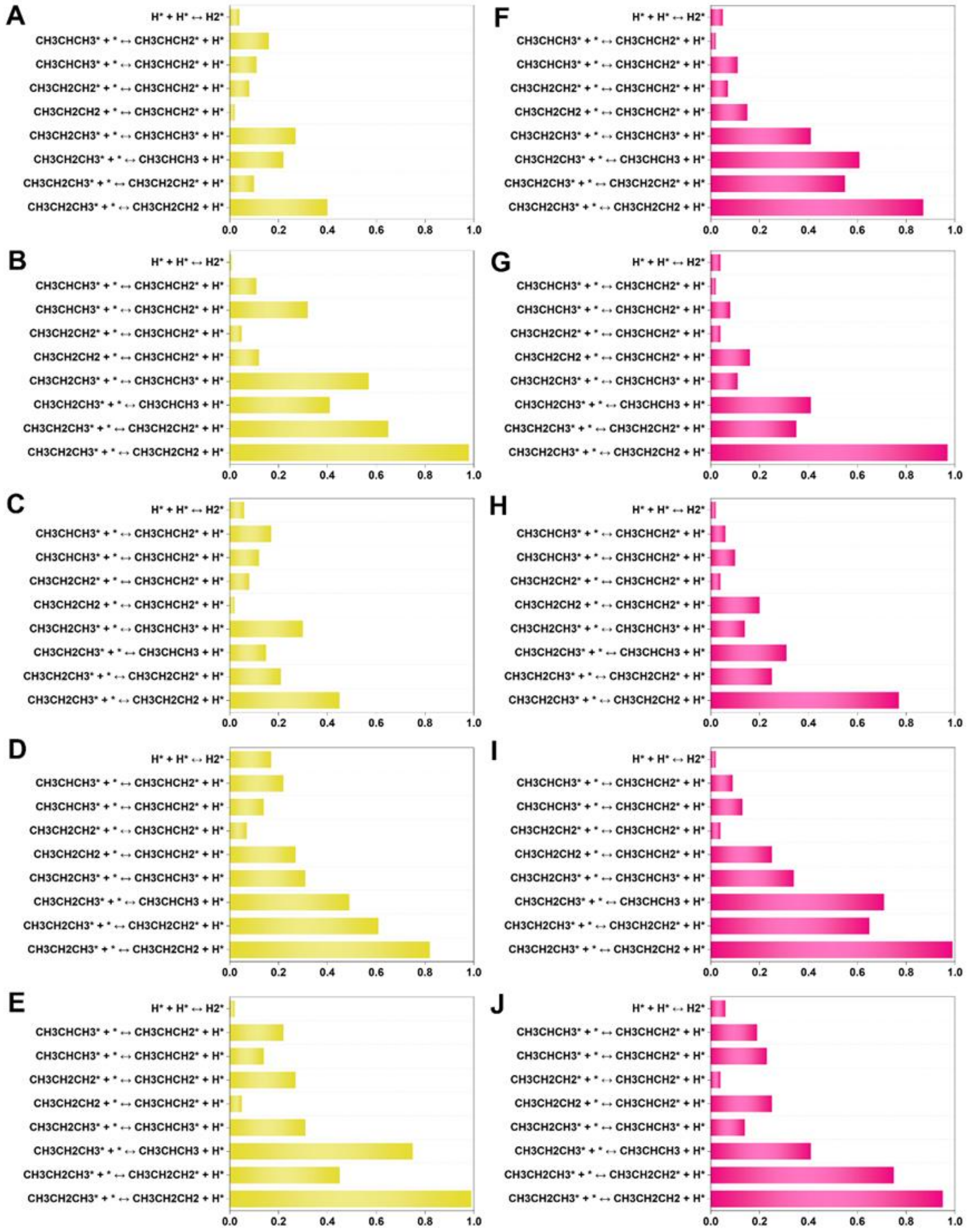

Figure S19. DRC coefficients (X_{RC}) for Arrhenius type elementary reactions in PDH at 800 K and 1 bar. (A-E) TM-C/N, and (F-J) TM-N/C, where TM = Ti, V, Nb, Mo, Ta, respectively, related to Figure 9.

Table S2. Computed lattice constants and relative stability of oxygen-functionalized MXene configurations, related to Figure 1.

MXenes	Type	Stable configuration	Lattice constant (Å)	MXenes	Type	Stable configuration	Lattice constant (Å)
TM-C	$Ti_3C_2O_2$	fcc-fcc	3.09	TM-C/N	Ti_3CNO_2	fcc-hcp	3.06
TM-C	$V_3C_2O_2$	fcc-fcc	2.91	TM-C/N	V_3CNO_2	fcc-fcc	2.90
TM-C	$Nb_3C_2O_2$	fcc-fcc	3.16	TM-C/N	Nb_3CNO_2	fcc-hcp	3.16
TM-C	$Mo_3C_2O_2$	hcp-hcp	3.13	TM-C/N	Mo_3CNO_2	hcp-fcc	3.19
TM-C	$Ta_3C_2O_2$	fcc-fcc	3.15	TM-C/N	Ta_3CNO_2	fcc-hcp	3.15
TM-N	$Ti_3N_2O_2$	fcc-fcc	3.05	TM-N/C	Ti_3CNO_2	fcc-hcp	3.06
TM-N	$V_3N_2O_2$	fcc-fcc	2.91	TM-N/C	V_3CNO_2	fcc-fcc	2.90
TM-N	$Nb_3N_2O_2$	fcc-fcc	3.18	TM-N/C	Nb_3CNO_2	fcc-hcp	3.16
TM-N	$Mo_3N_2O_2$	hcp-fcc	3.25	TM-N/C	Mo_3CNO_2	hcp-fcc	3.19
TM-N	$Ta_3N_2O_2$	fcc-fcc	3.19	TM-N/C	Ta_3CNO_2	fcc-hcp	3.15

Table S3. Comparison of the DFT calculated and ZPE-corrected barriers. First and second C-H bond activation energies (eV) during propane dehydrogenation on MXenes (TM-C, TM-N, TM-C/N and TM-N/C) for all reaction pathways; R1-M1, R1-M2, R2-M1, R2-M2, related to Figure 4.

Type	MXenes	DFT first barrier (eV)	DFT first barrier (eV)	DFT first barrier (eV)	DFT first barrier (eV)	ZPE corrected first barrier (eV)	ZPE corrected first barrier (eV)	ZPE corrected first barrier (eV)	ZPE corrected first barrier (eV)
		R1-M1	R1-M2	R2-M1	R2-M2	R1-M1	R1-M2	R2-M1	R2-M2
TM-C	$Ti_3C_2O_2$	0.81	1.25	1.36	1.46	0.78	1.23	1.34	1.42
TM-C	$V_3C_2O_2$	0.56	1.09	1.23	1.30	0.54	1.07	1.22	1.27
TM-C	$Nb_3C_2O_2$	1.11	1.69	1.82	1.82	1.07	1.66	1.79	1.79
TM-C	$Mo_3C_2O_2$	0.95	1.42	1.47	1.63	0.93	1.38	1.45	1.59
TM-C	$Ta_3C_2O_2$	1.21	1.86	1.92	2.06	1.17	1.84	1.89	2.05
TM-N	$Ti_3N_2O_2$	2.05	2.22	2.59	2.90	2.01	2.19	2.57	2.88
TM-N	$V_3N_2O_2$	1.83	1.95	2.32	2.70	1.79	1.92	2.30	2.67
TM-N	$Nb_3N_2O_2$	2.53	2.71	2.92	3.25	2.47	2.68	2.91	3.21
TM-N	$Mo_3N_2O_2$	2.38	2.42	2.76	3.17	2.33	2.39	2.73	3.13
TM-N	$Ta_3N_2O_2$	2.61	3.01	3.23	3.58	2.57	2.99	3.21	3.57
TM-C/N	Ti_3CNO_2	0.71	1.42	1.71	1.81	0.69	1.40	1.69	1.78
TM-C/N	V_3CNO_2	0.63	1.36	1.47	1.63	0.58	1.33	1.44	1.60
TM-C/N	Nb_3CNO_2	1.11	1.69	1.92	2.06	1.07	1.67	1.90	2.02
TM-C/N	Mo_3CNO_2	0.95	1.48	1.82	1.88	0.91	1.44	1.79	1.85
TM-C/N	Ta_3CNO_2	1.25	1.86	2.11	2.32	1.17	1.85	2.10	2.30
TM-N/C	Ti_3CNO_2	0.79	1.25	1.36	1.46	0.76	1.22	1.35	1.42
TM-N/C	V_3CNO_2	0.70	1.09	1.23	1.30	0.68	1.06	1.22	1.27
TM-N/C	Nb_3CNO_2	0.93	1.98	2.90	2.55	0.91	1.97	2.88	2.51
TM-N/C	Mo_3CNO_2	0.85	1.82	2.52	2.41	0.83	1.80	2.50	2.39
TM-N/C	Ta_3CNO_2	1.11	2.60	3.10	2.84	1.09	2.57	3.07	2.82
Type	MXenes	DFT second barrier (eV)	DFT second barrier (eV)	DFT second barrier (eV)	DFT second barrier (eV)	ZPE corrected second barrier (eV)	ZPE corrected second barrier (eV)	ZPE corrected second barrier (eV)	ZPE corrected second barrier (eV)
		R1-M1	R1-M2	R2-M1	R2-M2	R1-M1	R1-M2	R2-M1	R2-M2
TM-C	$Ti_3C_2O_2$	0.50	0.46	0.57	0.22	0.48	0.43	0.56	0.19
TM-C	$V_3C_2O_2$	0.47	0.41	0.56	0.21	0.45	0.40	0.54	0.20
TM-C	$Nb_3C_2O_2$	0.99	0.56	0.71	0.30	0.98	0.54	0.68	0.28
TM-C	$Mo_3C_2O_2$	0.68	0.50	0.63	0.26	0.64	0.47	0.61	0.23

TM-C	$Ta_3C_2O_2$	1.00	0.87	1.11	0.43	0.97	0.86	1.08	0.41
TM-N	$Ti_3N_2O_2$	0.50	0.48	0.37	0.60	0.47	0.46	0.34	0.57
TM-N	$V_3N_2O_2$	0.41	0.31	0.32	0.44	0.40	0.29	0.30	0.42
TM-N	$Nb_3N_2O_2$	0.61	0.64	0.55	1.11	0.59	0.62	0.51	1.09
TM-N	$Mo_3N_2O_2$	0.60	0.55	0.39	0.75	0.57	0.52	0.37	0.73
TM-N	$Ta_3N_2O_2$	0.69	0.94	0.97	2.00	0.66	0.93	0.98	1.97
TM-C/N	Ti_3CNO_2	0.51	0.72	0.59	0.64	0.50	0.70	0.56	0.63
TM-C/N	V_3CNO_2	0.50	0.69	0.55	0.29	0.49	0.66	0.53	0.27
TM-C/N	Nb_3CNO_2	0.83	0.91	0.93	0.71	0.81	0.89	0.90	0.68
TM-C/N	Mo_3CNO_2	0.57	0.85	0.79	0.68	0.55	0.84	0.77	0.66
TM-C/N	Ta_3CNO_2	1.31	0.92	1.11	1.41	1.28	0.90	1.09	1.39
TM-N/C	Ti_3CNO_2	0.82	0.86	1.01	0.42	0.80	0.83	0.98	0.40
TM-N/C	V_3CNO_2	0.76	0.77	0.62	0.40	0.74	0.75	0.60	0.38
TM-N/C	Nb_3CNO_2	1.58	1.46	1.37	1.14	1.56	1.44	1.36	1.11
TM-N/C	Mo_3CNO_2	1.16	1.32	1.42	0.53	1.15	1.31	1.40	0.51
TM-N/C	Ta_3CNO_2	1.62	1.57	2.07	1.55	1.59	1.55	2.04	1.51

Table S4. Elementary steps (R1 to R8) for propane dehydrogenation (PDH) on all MXenes. Ads., Des., and Form. denote adsorption, desorption, and formation, respectively. The reaction type is mentioned with respect to Scheme 1. Additional deep dehydrogenation steps (R9 to R12) are included for the optimal catalyst, $V_3N_2O_2$, related to Figures 8 and 9.

Reactions	Elementary reactions in MKMCXX format	Elementary reactions in standard format	Reaction type followed by Scheme 1
Ads.	CH3CH2CH3 + * → CH3CH2CH3*	C_3H_8 + * → C_3H_8*	
Ads.	CH3CHCH2 + * → CH3CHCH2*	C_3H_6 + * → C_3H_6*	
Ads.	H2 + * → H2*	H_2 + * → H_2*	
R1	CH3CH2CH3* + * → CH3CH2CH2 + H*	C_3H_8* + * → 1-C_3H_7 + H*	R1-M1
R2	CH3CH2CH3* + * → CH3CH2CH2* + H*	C_3H_8* + * → 1-C_3H_7* + H*	R1-M2
R3	CH3CH2CH3* + * → CH3CHCH3 + H*	C_3H_8* + * → 2-C_3H_7 + H*	R2-M1
R4	CH3CH2CH3* + * → CH3CHCH3* + H*	C_3H_8* + * → 2-C_3H_7* + H*	R2-M2
R5	CH3CH2CH2 + * → CH3CHCH2* + H*	1-C_3H_7 + * → 2-C_3H_6* + H*	R1-M1-R3
R6	CH3CH2CH2* + * → CH3CHCH2* + H*	1-C_3H_7* + * → 2-C_3H_6* + H*	R1-M2-R3
R7	CH3CHCH3 + * → CH3CHCH2* + H*	2-C_3H_7 + * → 2-C_3H_6* + H*	R2-M1-R4
R8	CH3CHCH3* + * → CH3CHCH2* + H*	2-C_3H_7* + * → 2-C_3H_6* + H*	R2-M2-R4
H_2 Form.	H* + H* → H2*	H* + H* → H_2*	Des.
Reactions (For $V_3N_2O_2$)	Elementary reactions in MKMCXX format (For $V_3N_2O_2$)	Elementary reactions in standard format (For $V_3N_2O_2$)	
R9	CH3CH2CH2* + * → CH3CH2CH* + H*	1-C_3H_7* + * → 2-C_3H_6* + H*	
R10	CH3CHCH3* + * → CH3CCH3* + H*	2-C_3H_7* + * → 1-C_3H_6* + H*	
R11	CH3CHCH2* → CH3CHCH* + *	2-C_3H_6* → 2-C_3H_5 + *	
R12	CH3CHCH2* → CH3CCH2* + *	2-C_3H_6* → 1-C_3H_5 + *	

Table S5. Catalytic performance of MXenes and conventional catalysts. PDH, ODH, and DDH indicate propane, oxidative, and direct dehydrogenation reactions, respectively. Deep dehydrogenation barriers, and reaction temperature are given in eV, and K, respectively, related to Figure 9.

Catalyst	Reaction Type	Reactant	Reaction Temperature (K)	Deep dehydrogenation barrier (eV)	Selectivity (%)	Target product	Ref.
$V_3N_2O_2$	PDH	Propane	> 650	> 1.8	100	C_3H_6	This work
Cr_2O_3	PDH	Propane	700-1000	0.92	60-70	C_3H_6	[S1]
PH_3-VO_x/Al_2O_x	PDH	Propane	883	/	~80	C_3H_6	[S2]
Ti_3AlC_2	ODH	n-butane	823	/	35, 25	Butene, Butadiene	[S3]
$Ti_3C_2T_x$	DDH	Ethylbenzene	823	0.21	97.5	Styrene	[S4]

Table S6. Comparison of energies between p(4×4) and p(5×5) supercells. E_{ads}, E_a, and ΔE represent adsorption, activation, and reaction energies, accordingly, related to STAR Methods.

Elementary reactions	P(4×4)	P(4×4)	P(4×4)	P(5×5)	P(5×5)	P(5×5)
	E_{ads} (eV)	E_a (eV)	ΔE (eV)	E_{ads} (eV)	E_a (eV)	ΔE (eV)
CH3CH2CH3 + * → CH3CH2CH3*	-0.207	-	-	-0.202	-	-
CH3CH2CH3* + * → CH3CHCH3* + H*	-	0.567	-1.725	-	0.563	-1.721
CH3CHCH3* + * → CH3CHCH2* + H*	-	1.114	-0.618	-	1.110	-0.616

Supplemental references

[S1] Huš, M., Kopač, D., Likozar, B. (2020). Kinetics of non-oxidative propane dehydrogenation on Cr_2O_3 and the nature of catalyst deactivation from first-principles simulations. J. Catal. 386, 126-138.

[S2] Gua, Y., Liu, H., Yang, M., Maa, Z., Zhao, L., Xing, W., Wu, P., Liu, X., Mintova, S., Bai, P., Yan, Z. (2020). Highly stable phosphine modified VO_x/Al_2O_3 catalyst in propane dehydrogenation. Appl. Catal. B: Environ. 274, 119089.

[S3] Ng, W. H., Gnanakumar, E. S., Batyrev, E., Sharma, S. K., Pujari, P. K., Greer, H. F., Zhou, W., Sakidja, R., Rothenberg, G., Barsoum, M. W., Shiju, N. R. (2018). The Ti_3AlC_2 MAX phase as an efficient catalyst for oxidative dehydrogenation of n-butane. Angew. Chem. 130, 1501-1506.

[S4] Diao, J., Hu, M., Lian, Z., Li, Z., Zhang, H., Huang, F., Li, B., Wang, X., Su, D. S., Liu, H. (2018). $Ti_3C_2T_x$ MXene catalyzed ethylbenzene dehydrogenation: active sites and mechanism exploration from both experimental and theoretical aspects. ACS Catal. 8, 10051-10057.
