# Supplementary material for: Tailoring the catalytic performance of MXenes in propane dehydrogenation by layer substitution from microkinetic simulations
Source: iScience. 2025 Sep 7;28(10):113480. doi: 10.1016/j.isci.2025.113480 (PMC12483649; doi:10.1016/j.isci.2025.113480)
Supplement: Data S1. Derivation of active site area and rotational parameters for microkinetic modeling, related to STAR Methods [file mmc2.pdf]

Data S1. Derivation of active site area and rotational parameters for microkinetic modeling, related to STAR Methods.

Lattice constant $a_{supercell}$ = 12.20 Å = 1.220 × 10^{-9} m
Lattice constant $b_{supercell}$ = 12.20 Å = 1.220 × 10^{-9} m
Area of supercell ($A_{supercell}$) is;
$A_{supercell} = a_{supercell} \times b_{supercell}$
$A_{supercell}$ = 1.220 × 10^{-9} × 1.220 × 10^{-9}
$A_{supercell}$ = 1.4884 × 10^{-18} m^2
However, propane adsorption typically occurs at a single active site (e.g., one surface O atom), not the entire supercell.

Active site area:

Each supercell contains multiple active sites (e.g., 16 O terminations for a 4×4 supercell). The effective area per active site is:

$$A_{site} = \frac{A_{supercell}}{Number\ of\ sites} = \frac{1.4884\ \times 10^{-18}\ m^2}{16} = 9.30\ \times\ 10^{-20}\ m^2$$

We rounded this to $1\ \times\ 10^{-19}\ m^2$ for simplicity, consistent with typical approximations for single-site adsorption in similar studies (e.g., Refs. [29, 30] in the manuscript).

Characteristic rotational temperature (θ_{rot}):

θ_{rot} represents the temperature at which thermal energy (k_BT) is comparable to the spacing between rotational energy levels. It is calculated as:

$$\theta_{rot} = \frac{hcB_e}{k_B}$$

H = Plank constant ($6.62\ \times\ 10^{-34}\ J.s$)
c = speed of light ($2.99\ \times\ 10^{10}\ cm/s$)
B_e = molecule's rotational onstant ($60.853\ cm^{-1}$)
k_B = Boltzmann constant ($1.38\ \times\ 10^{-23}\ J/K$)
For H_2 molecule, $\theta_{rot} = 85.3$ (ortho- H_2), and $\theta_{rot} = 64.2$ (para- H_2). At room temperature, ortho-H_2 dominates, therefore 85.3 K is typically used for kinetic modeling and simulations.
Propylene has an asymmetric top, therefore it has three principal rotational constants (A, B, and C); $A = 1.453$ K, $B = 0.744$ K, $C = 0.647$ K
The geometric mean of A, B and C is calculated as;

$$\theta_{rot} = \sqrt[3]{A.B.C}$$
$$\theta_{rot} = \sqrt[3]{1.453 \times 0.744 \times 0.647}$$
$$\theta_{rot} = 0.89\ K$$
